# Population-Specific Genetic Markers of Prostate Cancer Risk in Kazakh Men: Association Analysis of 102 SNPs and Risk Prediction Modeling

**DOI:** 10.3390/genes17080956

**Published:** 2026-08-14

**Authors:** Kairat Kazbekov, Yerbol Zhapparov, Nasrulla Shanazarov, Valery Benberin, Sergey Zinchenko, Ainagul Kazbekova

**Affiliations:** 1 Medical Center Hospital of the President’s Affairs Administration of the Republic of Kazakhstan, Astana 010000, Kazakhstan; 2 International Oncological Tomotherapy Center “Umit”, Astana 010000, Kazakhstan; 3 Department of Surgery, Kazan Federal University, Kazan 420008, Republic of Tatarstan, Russia; 4 Department of Internal Medicine, Astana Medical University, Astana 010000, Kazakhstan; kazbekova.a@amu.kz

**Keywords:** prostate cancer, single-nucleotide polymorphism, GWAS, Kazakh population, genetic risk of prostate cancer

## Abstract

Background/Objectives: GWASs have identified more than 250 prostate cancer (PCa) predisposition loci, predominantly in European and partly Asian cohorts. The Kazakh population is markedly under-represented in international genetic studies, limiting existing risk models. This study aimed to analyze the distribution of 102 PCa-associated single-nucleotide polymorphism (SNP) genotypes and alleles and to identify reliable population-specific associations with PCa risk in Kazakh men. Methods: This retrospective case–control study included 941 Kazakh men (476 with histologically confirmed PCa and 465 cancer-free controls). Genomic DNA extracted from peripheral blood was genotyped with TaqMan^®^ OpenArray^®^ technology on a QuantStudio 12K Flex system. Associations were assessed by Pearson’s χ^2^ test and logistic regression, with genotypic and allelic odds ratios (OR) and 95% confidence intervals (CI). Two-step multiple-testing correction (Bonferroni and Benjamini–Hochberg false-discovery rate, FDR) was applied. Predictive models were built using classification and regression trees (CART) and stepwise logistic regression. Results: Of 102 SNPs, 39 showed nominally significant genotypic differences; 12 remained significant after Bonferroni correction and 2 after FDR (14 in total). Several of the corrected loci were significant at both the genotypic and allelic level. Allelic ORs ranged from 0.37 (protective rs10187424 T allele) to 4.81 (rs1545985). A parsimonious seven-SNP autosomal logistic-regression model achieved an apparent AuROC of 0.84 (10-fold cross-validated 0.82); adding age as a covariate raised discrimination to 0.87. Ten of the fourteen significant loci remained significant after age adjustment, and six of these formed a core signal robust to both age imbalance and genotyping-quality concerns. Conclusions: This first large-scale SNP-association study in Kazakh men shows allele-frequency profiles resembling East Asian rather than European populations, confirming the need for population-specific genetic risk-assessment tools. The seven-SNP model showed high discriminatory power in the training set and requires external validation before clinical application.

## 1. Introduction

Prostate cancer (PCa) is one of the most commonly diagnosed cancers in men worldwide. According to GLOBOCAN 2022 data, approximately 1.47 million new patients with PCa and 397,000 deaths are recorded globally each year, making PCa a major public health issue in aging populations [1]. Kazakhstan’s epidemiological surveillance has registered a steady increase in the incidence of PCa over the last two decades, with a high proportion of late-stage cases (III–IV) and a five-year survival rate for metastatic disease not exceeding 34% [2,3]. This highlights an acute need to improve early risk stratification.

Genetic predisposition plays a vital role in the development of cancer. Based on twin and family study data, heritable (genetic) factors account for 40–60% of PCa risk variability [4,5]. Rare high-penetrance germline variants in DNA repair genes (BRCA2, ATM, CHEK2, PALB2) and the HOXB13 gene account for a clinically significant proportion of PCa patients, particularly those with aggressive or early-onset disease. However, they account for no more than 5–10% of all patients with PCa [6,7,8]. The remaining heritable risk is polygenic and mediated by the cumulative contribution of common SNPs, each exerting a moderate individual effect (OR typically 1.05–1.30) [9,10].

GWASs and trans-population meta-analyses have identified in excess of 250 independent susceptibility loci [9,11]. Region 8q24, which contains numerous enhancers that interact with the MYC oncogene, remains the most extensively studied [12,13]. The large trans-ancestry meta-analysis of Conti et al. consolidated these signals into a 269-variant polygenic risk score (PRS269), and subsequent multi-ancestry work has extended this to broader panels of several hundred variants; it is these GWAS-replicated loci that underpin currently used PRS [9,14,15]. The effectiveness of a PRS, however, depends on population origin: differences in allele frequencies, linkage disequilibrium (LD) patterns, and effect sizes can markedly reduce discriminatory power when European-derived models are applied to non-European populations [9,14,16]. Trans-population studies confirm that the effect sizes and allele frequencies of the same PCa risk SNPs vary substantially among European, African, and East Asian populations [15,17].

Central Asian populations, including the Kazakhs, are extremely under-represented in international genetic studies. The Kazakh population has a complex Eurasian origin, with genetic similarities to both European and East Asian groups, reflecting migration and the intermingling of peoples [18,19]. Of the 102 SNPs analyzed here, only one has been examined previously in the Kazakh population: the rs721048 (EHBP1) variant, whose association with PCa was recently reported locally [20]. The remaining loci are PCa susceptibility variants identified almost exclusively in European or East Asian GWASs [11,21,22] and have not been systematically investigated in Kazakh men; the great majority therefore remain untested in this population and warrant primary evaluation. The current understanding of the molecular-genetic markers of PCa has been summarized in a regional review [23].

This study addresses this gap through the first large-scale, population-based genetic study of prostate cancer in the Kazakh population—and the largest molecular-genetic investigation of this disease in Central Asia to date—analyzing 102 GWAS-derived or population-relevant SNPs in a case–control cohort of 941 men. Beyond this population, the work is directly relevant to the well-recognized Eurocentric bias of polygenic risk models and to the need for population-calibrated tools in under-represented groups. The main aim of the study is to identify SNPs that are significantly associated with PCa risk after two-stage adjustment for multiple comparisons. An additional objective is to determine whether the most informative SNPs can serve as the basis for a parsimonious predictive risk-stratification model for subsequent validation.

We identified 14 SNPs robustly associated with PCa risk—six of them significant after correction at both the genotypic and allelic level—and found that Kazakh allele-frequency profiles more closely resemble East Asian than European populations. A compact seven-SNP autosomal model achieved good discrimination (cross-validated AuROC ≈0.82, rising to ≈0.87 with age adjustment) in the training set, providing a first, population-calibrated step toward genetically informed, risk-stratified prostate cancer screening in a region with rising incidence and limited screening infrastructure, and contributing reference allele-frequency data for a population absent from major reference panels.

## 2. Materials and Methods

### 2.1. Study Design and Participants

The retrospective case–control study is based on archived clinical, demographic, and molecular-genetic data from the Scientific-Technical Programme of the Republic of Kazakhstan “Novel molecular-genetic approaches for pre-symptomatic diagnosis and treatment of the major diseases” (2017–2019). The analytical work was carried out between 2024 and 2026. Participating centers: Medical Center Hospital of the President’s Affairs Administration of the Republic of Kazakhstan and Multi-Disciplinary Medical Center of Astana City Mayor Office. Ethnicity was self-reported: all participants identified themselves as belonging to the Kazakh ethnic group. Self-reported ethnicity was not corroborated by genetic ancestry estimation (e.g., principal component analysis against reference panels); this is acknowledged as a limitation. The main group consisted of 476 patients with histologically confirmed PCa. The control group comprised 465 apparently healthy men with no medical history of cancer. For cases, the age variable corresponds to age at histological diagnosis; for controls, to age at enrolment/blood sampling. Age was recorded in the source archive for 794 of the 941 participants (84.4%: 340 PCa patients, 454 controls); the missing values reflect incomplete demographic fields in the retrospective archival records rather than any selective exclusion, and participants without a recorded age were retained in the genetic analyses. The report was prepared in accordance with the STROBE/STREGA guidelines [24,25].

Inclusion criteria, cases: men aged ≥18 years; histologically confirmed PCa; complete clinical and laboratory data; written informed consent in the archive records.

Inclusion criteria, controls: men aged ≥18 years; no medical history of cancer; written informed consent in the archive records.

Exclusion criteria (both groups): lack of histological verification (cases); incomplete or unreliable archive data; absence of written informed consent in the archive records; membership of vulnerable population groups. Records excluded for incomplete or unreliable data were distributed similarly between cases and controls and were removed before genotyping, so they are not expected to introduce differential selection.

Ethics: The study was conducted in accordance with the Declaration of Helsinki and was approved by the local bioethics committee of the RSE “Medical Center Hospital of the President’s Affairs Administration of the Republic of Kazakhstan” (Protocol No. 1, 9 February 2024). Written informed consent had been obtained from all participants at the time of the original sample and data collection (2017–2019), as documented in the archived records.

### 2.2. Samples, DNA Extraction, SNP Panel, and Genotyping

Biological samples and DNA extraction: Peripheral venous blood was collected from each participant into EDTA tubes during the 2017–2019 program and stored at −20 °C in the biobank. Genomic DNA was extracted from whole blood following a standardized in-house protocol aligned with EC FP7 procedures; DNA concentration and purity (A_260/280_) were assessed spectrophotometrically before genotyping.

An initial panel of 128 potential SNPs was selected based on three criteria: (a) significance in the GWAS catalogue (*p* < 5 × 10^−8^) in large-scale GWASs or cross-ethnic meta-analyses (≥10,000 participants) for the ‘prostate carcinoma’ phenotype; (b) confirmed association in East Asian cohorts (Japanese, Koreans) or previous pilot data from a Kazakh population [24,25,26]; (c) technical suitability for TaqMan^®^ OpenArray^®^ genotyping (validated probe design, MAF ≥ 0.01 according to dbSNP, absence of cross-hybridization in flanking regions). Following quality control checks for genotyping completeness (call rate ≥ 95%), with MAF ≥ 0.01 and HWE *p* > 0.001 in the control group (excluding known disease-associated loci), 102 SNPs were retained for analysis (Supplementary Tables S1 and S2).

Genotyping was performed using the TaqMan^®^ OpenArray^®^ Genotyping Plate Custom Format 128, OpenArray^®^ 384-well, and TaqMan^®^ OpenArray^®^ Genotyping Master Mix on the QuantStudio 12K Flex real-time PCR system (Applied Biosystems, Thermo Fisher Scientific, Waltham, MA, USA). Genotypes were determined through the QuantStudio 12K Flex v1.3 software in automatic mode, followed by manual verification of the clusters. Samples with call rates below 95% were excluded. Cases and controls were processed within the same project using a single genotyping protocol, reagent lot, and calling pipeline. Genotype clusters were re-examined manually while blinded to case–control status. Because cases and controls were not deliberately balanced across plates, a residual plate-level (batch) effect cannot be formally excluded; this is addressed as a limitation and the strongest signals are flagged for orthogonal re-genotyping.

### 2.3. Statistical Analysis

Hardy–Weinberg equilibrium (HWE) was tested in the control group only, using Pearson’s χ^2^ test; SNPs deviating from HWE in controls are interpreted with caution. Categorical variables were compared using Pearson’s χ^2^ test. Genotype distributions were analyzed with the common (major-allele) homozygote as the reference category, and genotypic odds ratios (OR) with 95% confidence intervals (CI) were computed for the heterozygous and variant-homozygous genotypes and for the dominant model. Independent multivariable effects were evaluated by logistic regression. Allele associations were assessed through a 2 × 2 contingency table, with allelic ORs and 95% CIs [27]. Effect estimates for loci showing a zero genotype cell (complete separation) were obtained with the Haldane–Anscombe correction and are flagged as unstable. To control for Type I errors when testing 102 SNPs simultaneously, the Bonferroni correction was applied with a threshold of *p* below 0.00049 (α/102 = 0.05/102). At the second level, FDR correction was performed utilizing the Benjamini–Hochberg method for *p* < 0.05. The results are presented in three categories: (a) SNPs significant in the Bonferroni test; (b) SNPs significant in FDR but not in Bonferroni; (c) nominally significant SNPs (*p* < 0.05) without correction for multiple comparisons. Statistical power was estimated for an allelic association model (chi-squared χ^2^ test with 1 degree of freedom; *n* = 476 PCa patients, *n* = 465 controls): for an OR of 1.5 and a minor-allele frequency (MAF) ≥ 0.10, the power exceeded 80% at *p* < 0.05; for OR ≥ 2.0 and MAF ≥ 0.10, the power exceeded 95% at the Bonferroni threshold; for rare variants (MAF < 0.05) with moderate effects (OR < 2.0), the power was < 55%.

Predictive models were constructed using CART (Classification and Regression Tree) and stepwise logistic-regression methods, with SNPs added based on the maximum increase in AuROC (stopping criterion: increase in AuROC < 0.001). Diagnostic performance was assessed using ROC analysis (AuROC, sensitivity, specificity, efficiency) at a probability threshold of *p* = 0.50. To address potential overfitting from developing and evaluating the model in the same sample, internal validation was performed using bootstrap optimism correction (Harrell’s method, 1000 resamples) and 10-fold cross-validation (20 repeats). Optimism-corrected discrimination is reported alongside the apparent AuROC. Prostate-specific antigen (PSA) data were not part of the model. The models were built exclusively from SNP genotypes; the PSA performance figures used for context were taken from a separately published PSA-based screening cohort of the Kazakh population [28] and were neither measured in the present participants nor entered into model training. All performance metrics—including the comparison with PSA specificity—are therefore apparent (training-set) estimates and require confirmation in an independent external cohort. Analyses were performed utilizing Statistica 10 (StatSoft Inc., Tulsa, OK, USA) and SAS JMP 11 (SAS Institute Inc., Cary, NC, USA).

## 3. Results

### 3.1. Study Population Characteristics

The genotyping cohort comprised 941 men of the Kazakh population: 476 PCa patients and 465 participants in the control group. Among participants with available age data (*n* = 794), PCa patients were significantly older than the control group (Me 64 years, IQR 56–71 vs. Me 53 years, IQR 45–61; *p* < 0.0001, Mann–Whitney U test). Men aged ≥60 years accounted for 67.1% of PCa patients, compared with 28.2% of the control group (χ^2^, *p* < 0.0001; Table 1).

### 3.2. Hardy–Weinberg Equilibrium

HWE was assessed in the control group only, as recommended for case–control designs. Of the 102 SNPs, 30 deviated from HWE in controls (*p* < 0.05), and these SNPs were included in further analysis only where appropriate and any significant results regarding these SNPs were noted. Several show a marked heterozygote excess in controls (for example rs10187424, with an observed heterozygote frequency well above Hardy–Weinberg expectation). All HWE results for controls are provided in Supplementary Table S3.

### 3.3. Ethnic Specificity of Allele Frequencies

Because the panel is dominated by variants originally discovered in European or East Asian GWASs, minor-allele frequencies (MAF) in the Kazakh control group were compared with 1000 Genomes Phase 3 reference populations [28] before presenting the association results (see Section 3.4 and Section 3.5), so that population differences can be taken into account when reading them (Table 2). Overall, the Kazakh allele-frequency profile corresponds more closely to East Asian than to European populations, consistent with the documented Eurasian mixed ancestry of the Kazakhs [18,19].

### 3.4. Genotypic Associations and Correction for Multiple Comparisons

Statistically significant genotypic differences (*p* < 0.05) were observed for 39 of the 102 SNPs (Supplementary Table S4). Genotype counts by group, with genotypic ORs and 95% CIs (common homozygote as reference), are given in Table 3; the numbers of successfully genotyped cases and controls are shown for each SNP so that per-locus genotyping completeness is transparent. After Bonferroni correction (*p* = 0.00049), 12 SNPs remained statistically significant: rs10187424, rs1545985, rs11228565, rs1469713, rs12621278, rs12418451, rs37181, rs10498792, rs7000448, rs13252298, rs1571801, and rs17361950 (Table 3). A further two loci, rs6465657 (*p* = 0.0006) and rs887391 (*p* = 0.0059), passed the FDR correction (*p* < 0.05), resulting in a total of 14 significant associations. Of the remaining 25 nominally significant SNPs (*p* = 0.001–0.050), up to five false positives are expected at a significance level of *p* < 0.05 based on the results of 102 tests; these results are considered preliminary until confirmed.

The X-linked locus rs5945572 (Xp11.22) is not reported as a genotypic association: outside the pseudoautosomal regions, males are hemizygous, so the diploid genotypic model does not apply and the heterozygous “calls” are not biologically valid. Under a locus-appropriate hemizygous (allelic) analysis, it showed no difference between groups (OR = 0.94; *p* = 0.59). It is therefore not treated as an associated marker.

### 3.5. Allelic Associations

In the allele-based analysis, significant associations were identified for 27 SNPs (*p* < 0.05). The strongest risk associations were identified for rs1545985 (allelic OR = 4.81; 95% CI: 3.67–6.31; *p* < 0.0001), rs1469713 (OR = 3.87; 95% CI: 2.85–5.25; *p* < 0.0001), rs11228565 (OR = 3.74; 95% CI: 2.91–4.81; *p* < 0.0001), rs1571801 (OR = 2.25; 95% CI: 1.61–3.14; *p* < 0.0001), and rs1529276 (OR = 2.12; 95% CI: 1.41–3.21; *p* = 0.0003). The most pronounced protective associations (allelic OR < 1.0) were observed for rs10187424 (major T allele; OR = 0.37; 95% CI: 0.30–0.45; *p* < 0.0001), rs17361950 (OR = 0.51; 95% CI: 0.32–0.80; *p* = 0.0029), rs12621278 (OR = 0.52; 95% CI: 0.37–0.72; *p* < 0.0001), rs10498792 (OR = 0.60; 95% CI: 0.44–0.80; *p* = 0.0006), and rs13252298 (OR = 0.65; 95% CI: 0.52–0.81; *p* < 0.0001) (Table 4).

### 3.6. Sensitivity Analyses: Age Adjustment and Hardy–Weinberg Consistency

Because cases were on average older than controls (mean 62.5 vs. 52.9 years), the 14 significant associations were re-examined with age as a covariate in an additive (minor-allele dosage) logistic model, restricted to the 794 participants with a recorded age (Supplementary Table S6). In total, 10 of the 14 loci remained significant after age adjustment; the effect sizes were essentially unchanged (for example rs10187424 OR 2.76 → 2.57; rs1469713 OR 5.29 → 5.02; rs887391 OR 0.64 → 0.56). rs10498792 and rs17361950 became borderline (*p* = 0.08 and 0.06), and rs6465657 and rs12418451 were not significant under the additive model.

Cross-classifying these results with control-group HWE identified a core of six loci that were both significant after age adjustment and in Hardy–Weinberg equilibrium in controls—rs11228565 (11q13.3), rs1571801 (DAB2IP), rs7000448 (CASC8), rs13252298 (AC020688.1), rs887391 (PCAT19), and rs1469713 (GATAD2A). This subset represents an association signal robust to both the age imbalance and the control-group HWE concern. A further four loci (rs10187424, rs1545985, rs12621278, rs37181) remained significant after age adjustment but showed HWE deviation in controls and are therefore interpreted with greater caution pending orthogonal re-genotyping.

### 3.7. Predictive Risk-Stratification Models

**Figure 1:**
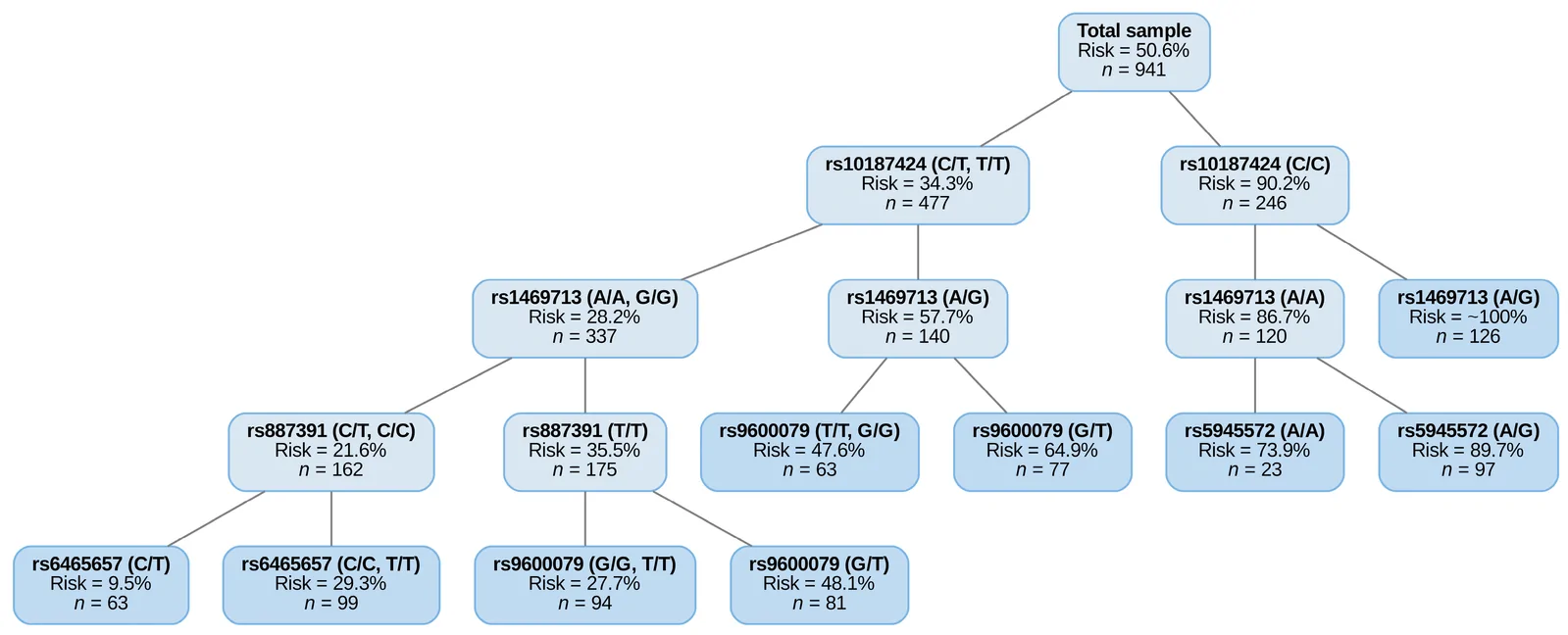
CART decision tree for prostate cancer risk stratification based on six SNPs (rs10187424, rs1469713, rs887391, rs9600079, rs5945572, rs6465657). The intensity of the blue color in the boxes represents the level of risk, ranging from light blue (lowest risk) to dark blue (highest risk).

Using the CART method, six SNPs defined the tree: rs10187424, rs1469713, rs5945572, rs9600079, rs887391, and rs6465657 (Figure 1). The highest-risk terminal category (rs10187424 C/C + rs1469713 A/G) had an observed PCa risk of 100.0% (*n* = 126; 17.4% of the 723 participants in the CART analysis). CART performance: AuROC = 0.86, sensitivity 77.8%, specificity 83.5%, accuracy 80.7% (full nine-class classification in Supplementary Table S7).

Each node shows the observed PCa risk (%) and number of participants (*n*). The root node corresponds to the full cohort (*n* = 941); 218 participants without an rs10187424 genotype call were excluded at the first split, and the tree below is based on the 723 participants with complete genotypes at all node markers. The highest-risk terminal group (rs10187424 C/C + rs1469713 A/G) reached ~100% observed risk (*n* = 126). Note that rs5945572 is retained here only as a data-driven predictor and is not a validated biological marker (Section 4.6).

Stepwise logistic regression selected eight SNPs over eight steps (Table 5). The univariate model (rs10187424 C/C) achieved an AuROC of 0.775; the largest single increment came from rs9600079 (ΔAuROC = +0.075). Although rs9600079 was only nominally significant in the genotypic analysis (χ^2^ = 7.60, *p* = 0.0224) and did not survive multiple-testing correction, it produced the largest independent increase in discrimination after the lead marker, i.e., its contribution is multivariable; it adds information jointly with the other predictors rather than as a strong single-marker effect.

The weighted-integer scoring model summarized in Table 5 and Table 6 reached an apparent AuROC of 0.88; when the same eight markers were refitted as a standard logistic regression with coefficients re-estimated within each cross-validation fold, the apparent discrimination was 0.85 and 0.82 on 10-fold cross-validation, indicating that the apparent 0.88 is optimistic (Table 6). Because rs5945572 is X-linked and its heterozygous calls in a male sample are not biologically valid, the model was refitted after removing this locus. Dropping rs5945572 changed discrimination only marginally (apparent AuROC 0.851 → 0.842; 10-fold cross-validated 0.826 → 0.817; ΔAuROC = 0.009), with sensitivity and specificity essentially unchanged. The seven-SNP model (rs10187424, rs9600079, rs1469713, rs1859962, rs12418451, rs887391, and rs6465657) is therefore adopted as the primary predictive model, and rs5945572 is not retained. Adding age as a covariate to this seven-SNP model raised discrimination to an apparent AuROC of 0.87 (10-fold cross-validated 0.85), consistent with the older age of the cases; a genotype-plus-age model is thus the best-performing combination, although all estimates are internal and require external validation.

The partial overlap between the CART and regression predictor sets is expected: CART optimizes individual binary splits, whereas stepwise logistic regression rewards complementary information in a multivariable context. rs9600079 and rs1859962 were only nominally significant individually and did not survive multiple-testing correction, yet both add independent variance to the multivariable model, while rs12418451 contributes a genotype-specific effect not captured by the key CART nodes.

## 4. Discussion

This study represents the most extensive systematic molecular-genetic analysis of PCa risk in the Kazakh male population (*n* = 941) and supports three main conclusions: (1) although only a small proportion of the panel—14 of the 102 analyzed SNPs (≈14%)—reached significance after correction for multiple comparisons, these loci replicate established GWAS signals in this previously unstudied Central Asian population; (2) allele frequencies and effect sizes differ substantially from European reference populations, more closely matching East Asian profiles; (3) a compact seven-SNP autosomal panel provides good discriminatory power in the training sample (apparent AuROC ≈ 0.84, 10-fold cross-validated ≈0.82; ≈0.87 with age), supporting the feasibility of a Kazakh-tailored genetic risk model that now requires external validation. Because all analyzed variants are germline (fixed at conception), reverse causation—disease or age altering genotype—is excluded; the observed age difference between groups is therefore addressed as a potential confounder acting through population stratification rather than as reverse causation (Section 4.6).

### 4.1. Interpretation of Key Associations

Before interpreting individual loci, one data-quality feature must be stated explicitly, because it conditions every association below. As is evident from the per-SNP denominators in Table 3, genotyping was markedly less complete in controls than in cases. Across the 14 corrected loci, the mean call rate was only ≈72% in controls versus ≈88% in cases, and for several loci, the number of successfully genotyped controls fell far below the ~400 that the design nominally provides, for example, ≈41% (rs37181), ≈49% (rs1545985), and ≈50% (rs10498792). Only three of the fourteen loci (rs10187424, rs11228565, rs1571801) retained ≥400 genotyped controls. This differential missingness is a genuine concern: unequal call rates between cases and controls can by themselves distort allele- and genotype-frequency comparisons and inflate apparent effect sizes, independently of any true biological association. It is also the most plausible explanation for the excess of Hardy–Weinberg departures in controls, since the loci that deviated from HWE had a substantially lower mean control call rate (≈64%) than those in equilibrium (≈82%)—that is, poor genotyping and HWE failure co-occur at the same loci. Associations resting on low control call rates should therefore be read as provisional and given less weight than the six core loci (rs11228565, rs1571801, rs7000448, rs13252298, rs887391, rs1469713), which combine control-group HWE consistency, robustness to age adjustment, and comparatively higher genotyping completeness. The individual loci discussed below are interpreted with this caveat throughout.

rs10187424 (VAMP8, 2p11.2) gave the strongest signal (χ^2^ = 281.15; C/C 8.5× more frequent in cases). rs10187424 at the VAMP8/GGCX locus is an established PCa susceptibility variant first reported in European GWASs and in near-complete LD (r^2^ ≈ 1) with the VAMP8 3′-UTR variant rs1058588. What is novel here is not the locus but the exceptional magnitude of its genotypic effect in the Kazakh population, which far exceeds previously reported effect sizes and therefore requires both technical confirmation and functional validation [22]; VAMP8 has additionally been implicated in tumor biology in other cancers [29]. The recessive/near-recessive pattern is consistent with population-specific LD with a functional variant that is rare in Europeans but common in Kazakhs. As this locus deviated from HWE in controls (Section 3.2), its effect estimate should be regarded as provisional until confirmed by orthogonal genotyping.

rs1545985 (FYCO1): FYCO1 encodes a Rab7 effector involved in autophagy and vesicular transport [30]. The G/G genotype was absent in controls (0%) but present in 26.8% of cases. Statistically, this is a situation of complete separation (a zero cell), which inflates the OR and its CI; the effect-size estimate for this locus is therefore provisional and should be verified by Sanger sequencing in an independent sample.

rs11228565 (11q13.3) was first described as a PCa risk marker in European cohorts, where the 11q13 signal was fine-mapped to rs11228565 [22]. In our cohort, it showed an allelic OR of 3.74. The comparable allele frequency in Kazakh controls (~11.8%) and East Asian populations (~10–15%), versus Europeans (~3–5%), reflects the ethnogenetic affinity of the Kazakh population with East Asian groups [18,19] rather than the locus’s discovery ancestry.

Protective variants: rs10187424 (T allele), rs12621278, rs17361950, rs10498792, rs13252298, rs887391, rs37181, and rs445114 showed protective allelic associations. Protective effects are a well-established feature of PCa GWASs and require no special biological justification; the relevant question is whether the direction of effect matches earlier reports. For the loci with prior GWAS evidence (e.g., rs12621278/ITGA6 and the 8q24/CASC region), the protective/risk directions observed here are broadly concordant with published European and East Asian GWASs, whereas the magnitudes are larger—consistent with population-specific LD. Loci without prior replication are reported as candidates for confirmation. rs37181 was significant under codominant, recessive, and overdominant but not dominant coding, a pattern compatible with heterozygote advantage that would require a functional study to confirm.

### 4.2. Ethnic Specificity and Implications for PRS

The allele-frequency comparison in Table 2 has direct implications for polygenic risk scoring. For 11 of the 14 corrected loci, the Kazakh minor-allele frequency lies closer to the East Asian than to the European 1000 Genomes reference, consistent with the documented Eurasian mixed ancestry of the Kazakhs. Because currently deployed polygenic risk scores are derived overwhelmingly from European cohorts, both their allele frequencies and their per-allele weights are poorly matched to a population with this East-Asian-leaning profile; such models are therefore expected to be miscalibrated and to lose discriminatory power when applied directly to Kazakh men. This provides the empirical rationale for developing and calibrating a population-specific PRS rather than importing European weights, and it should be tested formally against a European-derived PRS in an independent Kazakh validation cohort.

Validated European PRS models reach an AUC of 0.68–0.79 in their target populations [9]; applied across ancestry, the AUC can fall by up to five-fold [14,16]. A population-specific PRS for Kazakh men may therefore outperform European-derived models, although the present discrimination (apparent AuROC ≈ 0.84) was obtained in the training sample without external validation and is likely optimistic. The present data provide an empirical foundation for such a model; the immediate next steps are a prospective validation cohort and a PRS calibration study. Because our panel comprises variants discovered mainly in European datasets, it may miss loci that are polymorphic and PCa-associated specifically in East/Central Asian populations. A dedicated genome-wide association study in East Asian and Central Asian populations, including Kazakhs, is therefore warranted to discover novel, population-relevant risk variants that a Europe-derived panel cannot capture.

### 4.3. Clinical Implications

The compact seven-SNP model achieves high specificity (~81%), several-fold higher than PSA screening (10.4%) at the standard cut-off [27], at a sensitivity of ~75%; excluding the artefactual X-linked locus leaves this performance essentially unchanged. This comparison is indicative only: the PSA figures come from a separate cohort [27], were not measured in our participants, and did not enter the model, and our specificity is an apparent (training-set) estimate; a head-to-head comparison in the same prospective cohort is required. This profile would position the model as a supplementary second-line tool rather than a standalone screening test. PSA is more sensitive but drives many unnecessary biopsies, particularly in the 4–10 ng/mL ‘grey zone’, where only 20–25% of biopsies are positive; used as a reflex test here, a genetic model could in principle reduce unnecessary biopsies, similar to the Prostate Health Index (PHI) or 4Kscore [31,32]. Unlike protein markers, germline SNPs are stable throughout life, allowing stratification from an early age. On 10-fold cross-validation, the seven-SNP AuROC was ≈0.82 (≈0.85 with age), indicating some optimism in the apparent estimate; external validation remains essential.

### 4.4. Comparison with Published GWASs and PRS Studies

The observed effect sizes (allelic ORs up to 4.81) are considerably higher than typically reported in European GWASs (OR 1.1–1.5 for common SNPs) [9]. Possible explanations include population-specific stronger LD between the genotyped SNP and the causal variant, a true biological characteristic of this population, environmental differences, or enrichment of aggressive/familial cases. Without functional studies and independent replication, these cannot be distinguished. Cases and controls were genotyped within a single project using one protocol, reagent lot, and calling pipeline, with genotype clusters re-examined blinded to case–control status. However, we did not deliberately balance cases and controls across plates, and we did not Sanger sequence control samples to cross-check the automated calls; we can therefore not fully exclude a residual plate-level (batch) effect, and we explicitly flag this as a limitation. The largest effect sizes (and in particular the complete-separation loci rs1545985, rs1571801, and rs1469713 G/G) are treated as provisional pending orthogonal re-genotyping (Sanger/KASP) of both cases and controls in an independent subset. With these caveats, our results are consistent with trans-ethnic meta-analyses showing larger effect sizes for PCa loci in non-European, particularly East Asian, populations [9,15,17].

### 4.5. Methodological Considerations

HWE was evaluated in the control group only, and only control-group departures are interpreted. Of the 102 SNPs, 30 deviated from HWE in controls, including 8 of the 14 loci significant after correction (rs10187424, rs12621278, rs37181, rs10498792, rs1545985, rs17361950, rs6465657, and rs12418451). Associations at these loci are reported with explicit caution (Section 3.2). Such deviations may reflect extreme genotype enrichment, hidden population substructure [18], or platform-specific effects, for which formal interpretive frameworks exist [33]; the fact that they cluster among the most strongly associated loci raises the possibility that part of the signal is technical, and reinforces the need for orthogonal re-genotyping (Section 4.4). Principal component or STRUCTURE analysis would allow the influence of substructure to be quantified in future work. HWE data for controls are provided transparently (Supplementary Table S3) in accordance with STREGA [25].

### 4.6. Strengths and Limitations

Strengths: The largest genetic cohort of the Kazakh population to date (*n* = 941; 9–40× larger than previous Kazakh studies); a panel of 102 GWAS/East Asian/pilot SNPs; two-level correction for multiple comparisons (Bonferroni + FDR); complementary CART and logistic-regression models; power calculation for all effect sizes; transparent HWE reporting.

Limitations: First and most importantly, the retrospective design carries a substantial age imbalance between cases and controls (median 64 vs. 53 years; *p* < 0.0001). Because PCa risk rises steeply with age, and because age was missing for a proportion of participants and recorded as age at diagnosis in cases versus age at enrolment in controls, age is a serious potential confounder: unmatched groups can bias exposure–disease estimates, and matching or age adjustment would be required to ensure that observed allele-frequency differences are not driven by age structure. Although germline genotypes cannot themselves be altered by age (excluding reverse causation), age can still act as a confounder through latent population stratification if age cohorts differ in ethnogenetic composition, and the present predictive model was built on SNPs alone without age adjustment. We now explicitly acknowledge this as a substantial limitation that must be taken into account when interpreting the results; future analyses will incorporate age as a covariate and apply age-matching and formal control for population structure. Second, ethnicity was self-reported and not confirmed by genetic ancestry estimation; residual admixture or misclassification cannot be excluded, and genetic ancestry verification (e.g., PCA against reference panels) is planned. Third, genotyping was not orthogonally validated: no Sanger sequencing of control samples was performed to confirm the automated calls, and cases and controls were not balanced across plates, so a residual batch effect cannot be excluded—the strongest and complete-separation signals are consequently provisional. Fourth, rs5945572 (X-linked) initially entered the predictive model only as a data-driven predictor; because its heterozygous calls in a male sample are biologically invalid and most likely artefactual, the model was refitted without it (Section 3.7). Removal changed discrimination negligibly (ΔAuROC = 0.009), and the seven-SNP model without rs5945572 is therefore reported as primary. Further limitations: the absence of an external validation cohort (the cross-validated AuROC of ≈ 0.82 is likely optimistic relative to external performance; independent replication with *n* ≥ 300–400 is required); population substructure was not modeled (PCA/STRUCTURE not performed); most associated SNPs lie in non-coding regions, so their functional mechanisms (eQTL, enhancer activity) require experimental validation; clinical phenotyping depth was limited (Gleason score, PSA at diagnosis, and TNM stage were unavailable for many participants); and FDR-only associations (rs6465657, rs887391) are preliminary.

A further and important limitation concerns genotyping call rates, particularly in the control group. In most genome-wide association studies, only variants with a per-SNP call rate > 98% are retained for analysis, whereas in the present dataset, several loci fell well below this standard and, critically, did so asymmetrically—controls were genotyped less completely than cases (mean ≈72% vs. ≈88% across the corrected loci; as low as ~41% for rs37181). Such failure rates, and especially their imbalance between groups, can bias frequency estimates, inflate effect sizes, and generate spurious departures from Hardy–Weinberg equilibrium; the clustering of HWE deviations at the lowest-call-rate control loci suggests that part of the observed signal may be technical rather than biological. We report this openly so that readers can weight the findings accordingly. The strongest and complete-separation loci in particular require orthogonal re-genotyping (Sanger/KASP) of both cases and controls, and future work should apply a uniform high call-rate threshold (e.g., ≥98%) and balanced plate design before drawing firm conclusions.

## 5. Conclusions

This is, to our knowledge, the first large-scale, population-based genetic study of prostate cancer in the Kazakh population and the largest such investigation in Central Asia to date. In this case–control cohort of 941 men, 14 SNPs were confirmed after two-stage correction for multiple comparisons: 12 after Bonferroni (*p* = 0.00049) and 2 after FDR. Ten of the fourteen loci remained significant after adjustment for age, and six of these were additionally in Hardy–Weinberg equilibrium in controls (rs11228565, rs1571801, rs7000448, rs13252298, rs887391, and rs1469713), forming a core signal robust to both the age imbalance and genotyping-quality concerns. The seven-SNP logistic-regression model achieved an apparent AuROC of 0.84 (10-fold cross-validated 0.82) in the training sample, rising to 0.87 when age was included as a covariate; ten of the fourteen significant loci, including six that were also in HWE in controls, remained significant after age adjustment. No clinical use should be attempted until independent external replication and calibration have been completed. Allele frequencies in the Kazakh population correspond to East Asian rather than European populations, confirming the need for population-adapted genetic tools. The largest effect sizes rest partly on complete-separation loci and on genotyping that has not yet been orthogonally validated, and the groups differ substantially in age; these findings should therefore be regarded as hypothesis-generating until confirmed.

By providing the first population-scale characterization of prostate cancer risk-allele architecture in Kazakh men, these data offer an empirical basis for population-specific polygenic risk scores and help reduce the Eurocentric bias of current models. Replication in an independent Kazakh cohort, orthogonal genotyping of the strongest signals, age-adjusted and ancestry-controlled modeling, and a dedicated GWAS in Central/East Asian populations are the priority next steps.

## Figures and Tables

**Table 1:** Baseline characteristics of the study population.

Characteristic	Total	Case, (*n* = 476)	Control, (*n* = 465)	*p* Value
Total genotyped, *n*	941	476	465	-
Age available, *n* (%)	794 (84.4%)	340 (71.4%)	454 (97.6%)	<0.0001 ^1^
Age, median (IQR), years	57 (48–68)	64 (56–71)	53 (45–61)	
Age range, years	18–88	22–86	18–88	-
Age <60 years, *n* (%)	438 (55.2%)	112 (32.9%)	326 (71.8%)	<0.0001 ^1^
Age ≥60 years, *n* (%)	356 (44.8%)	228 (67.1%)	128 (28.2%)	<0.0001 ^1^

^1^ Statistically significant at *p* < 0.05 (Mann–Whitney U test for continuous variables; chi-square χ^2^ for categorical variables). Note: IQR = interquartile range. Age data are available for 794 out of 941 participants (84.4%).

**Table 2:** Minor-allele frequencies (MAF) in Kazakh controls versus 1000 Genomes Phase 3 reference populations (EUR, European; EAS, East Asian) for the 14 SNPs significant after correction. The final column indicates the reference population to which the Kazakh frequency is numerically closer.

SNP (Gene)	Minor Allele	Kazakh Controls	EUR	EAS	Closer to
rs10187424 (VAMP8)	C	0.397	0.443	0.386	EAS
rs1545985 (FYCO1)	G	0.196	0.349	0.004	EUR
rs11228565 (11q13.3)	A	0.118	0.214	0.047	EAS
rs12621278 (ITGA6)	G	0.157	0.039	0.267	EAS
rs37181 (COMMD10)	G	0.394	0.228	0.455	EAS
rs10498792 (PKHD1)	C	0.225	0.105	0.135	EAS
rs1571801 (DAB2IP)	T	0.067	0.255	0.063	EAS
rs17361950 (FAAH)	T	0.074	0.270	0.018	EAS
rs7000448 (CASC8)	T	0.252	0.405	0.266	EAS
rs13252298 (AC020688.1)	G	0.424	0.288	0.356	EAS
rs6465657 (LMTK2)	T	0.278	0.510	0.150	EAS
rs887391 (PCAT19)	C	0.297	0.227	0.390	EUR
rs1469713 (GATAD2A)	G	0.080	0.337	0.320	EAS
rs12418451 (AP003071.2)	A	0.269	0.299	0.029	EUR

Note: Kazakh control MAF were computed directly from the study genotypes; EUR and EAS frequencies are 1000 Genomes Phase 3 values (Ensembl) for the same minor allele. For 11 of the 14 loci, the Kazakh frequency lies closer to the East Asian than to the European reference; clear East-Asian-like patterns (minor allele much rarer than in Europeans) are seen at rs17361950 (FAAH), rs1571801 (DAB2IP), rs6465657 (LMTK2), and rs11228565 (11q13.3), whereas rs12621278 (ITGA6) shows the opposite direction (allele rarer in Europeans than in Kazakhs).

**Table 3:** Genotypic associations for the 14 SNPs significant after multiple-testing correction (12 Bonferroni, 2 FDR). Genotypes: common-homozygote (reference)/heterozygote/variant-homozygote.

SNP (Gene)-Total Genotyped	Genotype	Control *n* (%)	Case *n* (%)	OR (95% CI)	*p* ^1^
rs10187424 (VAMP8) ^3^-Co 421/Ca 454 [Bonf.]	T/T (ref)	114 (27.1)	121 (26.7)	1.00 (ref)	<0.0001
	C/T	280 (66.5)	85 (18.7)	0.29 (0.20–0.41)	
	C/C	27 (6.4)	248 (54.6)	8.65 (5.40–13.88)	
rs1545985 (FYCO1) ^3^-Co 227/Ca 388 [Bonf.]	A/A (ref)	138 (60.8)	73 (18.8)	1.00 (ref)	<0.0001
	A/G	89 (39.2)	211 (54.4)	4.48 (3.08–6.53)	
	G/G ^2^	0 (0.0)	104 (26.8)	393.8 (24.1–6430) ^2^	
rs11228565 (11q13.3)-Co 427/Ca 440 [Bonf.]	G/G (ref)	334 (78.2)	200 (45.5)	1.00 (ref)	<0.0001
	A/G	85 (19.9)	186 (42.3)	3.65 (2.68–4.99)	
	A/A	8 (1.9)	54 (12.3)	11.27 (5.26–24.17)	
rs1469713 (GATAD2A)-Co 376/Ca 432 [Bonf.]	A/A (ref)	316 (84.0)	217 (50.2)	1.00 (ref)	<0.0001
	A/G	60 (16.0)	213 (49.3)	5.17 (3.70–7.22)	
	G/G ^2^	0 (0.0)	2 (0.5)	7.28 (0.35–152) ^2^	
rs12621278 (ITGA6) ^3^-Co 309/Ca 410 [Bonf.]	A/A (ref)	249 (80.6)	342 (83.4)	1.00 (ref)	<0.0001
	A/G	23 (7.4)	64 (15.6)	2.03 (1.22–3.35)	
	G/G	37 (12.0)	4 (1.0)	0.08 (0.03–0.22)	
rs12418451 (AP003071.2) ^3^-Co 342/Ca 428 [Bonf.]	G/G (ref)	237 (69.3)	274 (64.0)	1.00 (ref)	<0.0001
	A/G	26 (7.6)	95 (22.2)	3.16 (1.98–5.04)	
	A/A	79 (23.1)	59 (13.8)	0.65 (0.44–0.94)	
rs37181 (COMMD10) ^3^-Co 189/Ca 353 [Bonf.]	T/T (ref)	89 (47.1)	164 (46.5)	1.00 (ref)	<0.0001
	G/T	51 (27.0)	158 (44.8)	1.68 (1.12–2.53)	
	G/G	49 (25.9)	31 (8.8)	0.34 (0.20–0.58)	
rs10498792 (PKHD1) ^3^-Co 233/Ca 382 [Bonf.]	T/T (ref)	159 (68.2)	277 (72.5)	1.00 (ref)	<0.0001
	C/T	43 (18.5)	97 (25.4)	1.29 (0.86–1.95)	
	C/C	31 (13.3)	8 (2.1)	0.15 (0.07–0.33)	
rs1571801 (DAB2IP)-Co 403/Ca 453 [Bonf.]	G/G (ref)	349 (86.6)	343 (75.7)	1.00 (ref)	<0.0001
	G/T	54 (13.4)	94 (20.8)	1.77 (1.23–2.55)	
	T/T ^2^	0 (0.0)	16 (3.5)	33.6 (2.0–562) ^2^	
rs17361950 (FAAH) ^3^-Co 336/Ca 420 [Bonf.]	C/C (ref)	301 (89.6)	387 (92.1)	1.00 (ref)	<0.0001
	C/T	20 (6.0)	33 (7.9)	1.28 (0.72–2.28)	
	T/T ^2^	15 (4.5)	0 (0.0)	0.03 (0.00–0.42) ^2^	
rs7000448 (CASC8)-Co 391/Ca 441 [Bonf.]	C/C (ref)	222 (56.8)	203 (46.0)	1.00 (ref)	0.0004
	C/T	141 (36.1)	174 (39.5)	1.35 (1.01–1.81)	
	T/T	28 (7.2)	64 (14.5)	2.50 (1.54–4.05)	
rs13252298 (AC020688.1)-Co 297/Ca 406 [Bonf.]	A/A (ref)	94 (31.6)	179 (44.1)	1.00 (ref)	0.0003
	A/G	154 (51.9)	192 (47.3)	0.65 (0.47–0.91)	
	G/G	49 (16.5)	35 (8.6)	0.38 (0.23–0.62)	
rs6465657 (LMTK2) ^3^-Co 325/Ca 421 [FDR]	C/C (ref)	159 (48.9)	219 (52.0)	1.00 (ref)	0.0006
	C/T	151 (46.5)	154 (36.6)	0.74 (0.55–1.00)	
	T/T	15 (4.6)	48 (11.4)	2.32 (1.26–4.30)	
rs887391 (PCAT19)-Co 392/Ca 444 [FDR]	T/T (ref)	192 (49.0)	264 (59.5)	1.00 (ref)	0.0059
	C/T	167 (42.6)	157 (35.4)	0.68 (0.51–0.91)	
	C/C	33 (8.4)	23 (5.2)	0.51 (0.29–0.89)	

Note: ^1^ Overall genotypic χ^2^ *p*-value for the SNP. ^2^ Genotype cell of zero (complete separation): the OR/CI were obtained with the Haldane–Anscombe correction and are unstable—these estimates are provisional pending orthogonal re-genotyping. ^3^ Several of these loci (rs10187424, rs12621278, rs37181, rs10498792, rs1545985, rs17361950, rs6465657, and rs12418451) deviated from HWE in controls and their associations should be read with the corresponding caution. Percentages are of successfully genotyped participants in each group; denominators (“Co”—controls, “Ca”—cases) differ between SNPs owing to variable genotyping completeness. Dominant-model ORs are given in the text where informative.

**Table 4:** Allelic associations for key SNPs.

SNP	Minor Allele	Case, *n* (%)	Control, *n* (%)	OR (95% CI)	*p* Value
rs10187424	T	327 (36.0)	508 (60.3)	0.37 (0.30–0.45)	0.0001
rs1545985	G	419 (54.0)	89 (19.6)	4.81 (3.67–6.31)	0.0001
rs11228565	A	294 (33.4)	101 (11.8)	3.74 (2.91–4.81)	0.0001
rs1469713	G	217 (25.1)	60 (8.0)	3.87 (2.85–5.25)	0.0001
rs12621278	G	72 (8.8)	97 (15.7)	0.52 (0.37–0.72)	0.0001
rs37181	G	220 (31.2)	149 (39.4)	0.70 (0.54–0.90)	0.0063
rs10498792	C	113 (14.8)	105 (22.5)	0.60 (0.44–0.80)	0.0006
rs1571801	T	126 (13.9)	54 (6.7)	2.25 (1.61–3.14)	0.0001
rs17361950	T	33 (3.9)	50 (7.4)	0.51 (0.32–0.80)	0.0029
rs7000448	T	302 (34.2)	197 (25.2)	1.55 (1.25–1.91)	0.0001
rs13252298	G	262 (32.3)	252 (42.4)	0.65 (0.52–0.81)	0.0001
rs887391	C	203 (22.9)	233 (29.7)	0.70 (0.56–0.87)	0.0014
rs1529276	A	114 (15.2)	32 (7.8)	2.12 (1.41–3.21)	0.0003
rs817826	C	92 (11.1)	42 (7.0)	1.67 (1.14–2.44)	0.0082
rs17041869	G	104 (13.9)	46 (9.7)	1.50 (1.04–2.16)	0.0312
rs10492519	G	232 (26.9)	152 (20.0)	1.47 (1.16–1.85)	0.0012
rs1016343	T	193 (22.1)	124 (16.8)	1.40 (1.09–1.80)	0.0085
rs6545977	A	432 (49.8)	310 (42.1)	1.36 (1.12–1.66)	0.0022
rs4466137	T	209 (29.1)	96 (23.2)	1.36 (1.03–1.80)	0.0306
rs1859962	G	450 (52.1)	343 (47.0)	1.23 (1.01–1.49)	0.0426
rs17021918	T	250 (27.8)	255 (32.3)	0.81 (0.66–1.00)	0.0469
rs6738028	G	347 (42.4)	286 (48.1)	0.79 (0.64–0.98)	0.0326
rs2121875	C	279 (36.9)	205 (42.9)	0.78 (0.62–0.98)	0.0360
rs11214775	A	212 (28.6)	150 (34.4)	0.77 (0.59–0.99)	0.0389
rs445114	C	257 (44.0)	301 (50.7)	0.77 (0.61–0.96)	0.0220
rs7501939	T	310 (37.0)	285 (43.7)	0.76 (0.61–0.93)	0.0086
rs2735839	A	205 (22.4)	259 (30.2)	0.67 (0.54–0.83)	0.0002

Note: Allele counts and frequencies are calculated from the number of successfully genotyped alleles at each locus; denominators therefore differ between SNPs owing to variable genotyping completeness (Supplementary Table S5).

**Table 5:** Stepwise logistic regression: order of predictor inclusion and final model performance.

Step	SNP (Risk Genotype)	AuROC	ΔAuROC	Weighted Score	*p* Value
1	rs10187424 (C/C)	0.775	+0.275	37	<0.0001
2	rs9600079 (G/T)	0.850	+0.075	7	0.0018
3	rs1469713 (A/G)	0.858	+0.008	10	<0.0001
4	rs1859962 (G/G)	0.864	+0.006	7	0.0037
5	rs12418451 (A/G)	0.871	+0.007	8	0.0063
6	rs887391 (T/T)	0.875	+0.004	7	0.0015
7	rs5945572 (A/G)	0.880	+0.005	15	0.0046
8	rs6465657 (T/T)	0.881	+0.001	9	0.0221
Final model (Regression 8)		AuROC 0.88	Cut-off: ≥24 pts	Sens. 70.64%	Spec. 86.33%

Note: Weighted score = proportional integer coefficient from the logistic regression, normalized to 0–100. Stopping criterion: increase in AuROC < 0.001. Accuracy assessment on the overall cohort (*n* = 941). Internal validation indicated minimal optimism: after bootstrap correction (1000 resamples) the AuROC decreased from 0.88 (apparent) to 0.87 (optimism 0.007), and 10-fold cross-validation of this weighted-score model gave 0.87 ± 0.04, whereas a standard logistic refit with per-fold coefficient re-estimation gave 0.82. rs5945572 (X-linked) entered at step 7 of the stepwise selection; because its heterozygous calls in a male sample are artefactual, the model was refitted without it, which changed AuROC by only 0.009. The seven-SNP model without rs5945572 is therefore adopted as the primary model. All metrics are apparent (training-set) estimates and require external validation.

**Table 6:** Comparative diagnostic performance of predictive models.

Model	Cut-Off	AuROC	Sensitivity	Specificity	Accuracy
CART decision tree	48.1%	0.86	77.8%	83.5%	80.7%
Regression 8 (preferred)	≥24 pts	0.88	70.64%	86.33%	78.48%
Regression 7	≥25 pts	0.88	70.08%	86.33%	78.21%
Regression 6	≥21 pts	0.88	80.33%	76.17%	78.25%
Score 8 (equal weights)	≥2	0.85	88.09%	54.69%	71.39%
PSA ≥ 4 ng/mL (reference)	4 ng/mL	-	96.6%	10.4%	-

Note: Regression 8 has the highest specificity (86.33%). The PSA figures derive from a separate published Kazakh screening cohort [27] and were not measured in the present sample; the comparison is therefore indicative only and both estimates are apparent. Sample sizes differ across stages owing to genotype completeness: CART used 723 participants; the final logistic regression was fitted to 617 participants with complete genotypes across all 8 SNPs.

## Data Availability

The genotyping and clinical datasets generated and analyzed during the current study are not publicly available and cannot be shared due to the confidentiality requirements of the original state research program and national personal-data protection legislation.

## Supplementary Materials

The following supporting information can be downloaded at https://www.mdpi.com/article/10.3390/genes17080956/s1. Table S1: Characteristics of all 102 analyzed SNPs; Table S2: Genotype distributions in controls and cases (all 102 SNPs); Table S3: Hardy–Weinberg equilibrium in the control group (all 102 SNPs); Table S4: Genotypic associations for SNPs significant by genotype distribution (χ^2^, *p* < 0.05); Table S5: Allelic associations for all 102 SNPs (minor-allele odds ratios); Table S6: Age-adjusted associations for the 14 significant SNPs (sensitivity analysis); Table S7: Risk classes identified using the decision tree method.

## Abbreviations

The following abbreviations are used in this manuscript:

AUC	Area under the curve
AuROC	Area under the receiver operating characteristic curve
CART	Classification and regression tree
CI	Confidence interval
DNA	Deoxyribonucleic acid
eQTL	Expression quantitative trait locus
FDR	False discovery rate
GLOBOCAN	Global Cancer Observatory database (IARC estimates of cancer incidence and mortality)
GWAS	Genome-wide association study
HWE	Hardy–Weinberg equilibrium
IQR	Interquartile range
LD	Linkage disequilibrium
MAF	Minor allele frequency
Me	Median
OR	Odds ratio
PCA	Principal component analysis
PCa	Prostate cancer
PCR	Polymerase chain reaction
PHI	Prostate Health Index
PRS	Polygenic risk score
PSA	Prostate-specific antigen
ROC	Receiver operating characteristic
RR	Relative risk
SNARE	Soluble N-ethylmaleimide-sensitive factor attachment protein receptor
SNP	Single-nucleotide polymorphism
STREGA	Strengthening the Reporting of Genetic Association Studies
STROBE	Strengthening the Reporting of Observational Studies in Epidemiology
TNM	Tumor, Node, Metastasis (classification)
