# Supplementary material for: Population-Specific Genetic Markers of Prostate Cancer Risk in Kazakh Men: Association Analysis of 102 SNPs and Risk Prediction Modeling
Source: Genes (Basel). 2026 Aug 14;17(8):956. doi: 10.3390/genes17080956 (PMC13512394; doi:10.3390/genes17080956)
Supplement: Supplementary file 1 [file genes-17-00956-s001.zip › genes-4446115-supplementary.pdf]

Table S1. **Characteristics of all 102 analysed SNPs**

| № | SNP (rsID) | Gene/locus | Chr | Position | Reference allele | Minor allele | MAF |
|---|---|---|---|---|---|---|---|
| **1** | **2** | **3** | **4** | **5** | **6** | **7** | **8** |
| 1 | rs76934034 | | 10 | 45587537 | T | C | 0.053 |
| 2 | rs10009409 | | 4 | 72989536 | C | T | 0.392 |
| 3 | rs684232 | VPS53 | 17 | 618965 | T | C | 0.417 |
| 4 | rs1016343 | CASC19 | 8 | 128093297 | C | T | 0.168 |
| 5 | rs74315365 | RNASEL | 1 | 182586804 | C | T | 0.003 |
| 6 | rs10492519 | FAM124A | 13 | 51250192 | A | G | 0.200 |
| 7 | rs13385191 | LDAH | 2 | 20688505 | A | G | 0.452 |
| 8 | rs2987983 | ESR2 | 14 | 64296935 | A | G | 0.312 |
| 9 | rs12155172 | LINC01162 | 7 | 20954872 | A | G | 0.725 |
| 10 | rs12827748 | PAWR | 12 | 79694798 | C | T | 0.419 |
| 11 | rs10498792 | PKHD1 | 6 | 51801833 | T | C | 0.225 |
| 12 | rs11650494 | AC004797.1 | 17 | 49267824 | G | A | 0.062 |
| 13 | rs6465657 | LMTK2 | 7 | 98187015 | C | T | 0.278 |
| 14 | rs121908982 | MAD1L1 | 7 | 2225526 | G | A | 0.002 |
| 15 | rs12418451 | AP003071.2 | 11 | 68935419 | G | A | 0.269 |
| 16 | rs345013 | | 3 | 145456001 | A | G | 0.172 |
| 17 | rs7652331 | FYCO1 | 3 | 45921260 | T | C | 0.796 |
| 18 | rs10187424 | VAMP8 | 2 | 85567174 | T | C | 0.397 |
| 19 | rs13149290 | ZNF827 | 4 | 145829814 | C | T | 0.523 |
| 20 | rs3217869 | CCND2 | 12 | 4290804 | G | A | 0.669 |
| 21 | rs2660753 | 3p12 | 3 | 87061524 | T | C | 0.746 |
| 22 | rs17361950 | FAAH | 1 | 46398478 | C | T | 0.074 |
| 23 | rs887391 | PCAT19 | 19 | 41985624 | T | C | 0.297 |
| 24 | rs5919432 | AR | X | 67801708 | C | T | 0.916 |
| 25 | rs6545977 | OTX1 | 2 | 63074029 | G | A | 0.421 |
| 26 | rs4775302 | AC091074.1 | 15 | 46347610 | G | A | 0.504 |
| 27 | rs2238135 | AC121338.1 | 12 | 47884407 | C | G | 0.287 |
| 28 | rs7679673 | AC004069.1 | 4 | 105140377 | C | A | 0.625 |
| 29 | rs817826 | KLF4 | 9 | 107394019 | C | T | 0.930 |
| 30 | rs9311171 | CTDSPL | 3 | 37954986 | T | G | 0.136 |
| 31 | rs9600079 | KLF5 | 13 | 73154002 | G | T | 0.446 |
| 32 | rs1456315 | AC020688.1 | 8 | 127091692 | T | C | 0.566 |
| 33 | rs137853009 | CHEK2 | 22 | 28725030 | C | T | 0.166 |

| | | | | | | | |
|---|---|---|---|---|---|---|---|
| 34 | rs17041869 | BCL2L11 | 2 | 111138666 | A | G | 0.097 |
| 35 | rs1447295 | CASC8 | 8 | 127472793 | A | C | 0.853 |
| 36 | rs7127900 | TH | 11 | 2212344 | A | G | 0.770 |
| 37 | rs119484087 | ELAC2 | 17 | 12995006 | T | A | 0.004 |
| 38 | rs7153648 | SIX1 | 14 | 60655808 | C | G | 0.874 |
| 39 | rs7501939 | HNF1B | 17 | 36101156 | C | T | 0.437 |
| 40 | rs10934853 | EEFSEC | 3 | 128319530 | C | A | 0.366 |
| 41 | rs6983267 | CASC8 | 8 | 127401060 | G | T | 0.473 |
| 42 | rs629242 | KIAA1211 | 4 | 56276871 | C | T | 0.272 |
| 43 | rs17599629 | GOLPH3L | 1 | 150685811 | A | G | 0.216 |
| 44 | rs121909139 | KLF6 | 10 | 3781971 | A | G | 0.152 |
| 45 | rs4430796 | HNF1B | 17 | 36098040 | A | G | 0.409 |
| 46 | rs37181 | COMMD10 | 5 | 116294307 | T | G | 0.394 |
| 47 | rs12500426 | PDLIM5 | 4 | 95514609 | A | C | 0.484 |
| 48 | rs121908701 | CHEK2 | 22 | 28725027 | C | C | 1.000 |
| 49 | rs12621278 | ITGA6 | 2 | 172446825 | A | G | 0.335 |
| 50 | rs4466137 | HAPLN1 | 5 | 83689920 | T | G | 0.555 |
| 51 | rs10993994 | MSMB | 10 | 46046326 | A | G | 0.157 |
| 52 | rs11228565 | 11q13.3 | 11 | 68978580 | G | A | 0.082 |
| 53 | rs5945572 | NUDT11 | X | 51486831 | A | G | 0.097 |
| 54 | rs10896449 | MYEOV | 11 | 68994667 | A | G | 0.344 |
| 55 | rs11214775 | HTR3B | 11 | 113936459 | G | A | 0.531 |
| 56 | rs4245739 | MDM4 | 1 | 204549714 | C | A | 0.477 |
| 57 | rs2292884 | MLPH | 2 | 237534583 | A | G | 0.159 |
| 58 | rs1545985 | FYCO1 | 3 | 45963986 | G | A | 0.804 |
| 59 | rs4054823 | | 17 | 13721707 | T | C | 0.548 |
| 60 | rs2121875 | FGF10 | 5 | 44365443 | C | A | 0.571 |
| 61 | rs636291 | PEX14 | 1 | 10496040 | G | A | 0.657 |
| 62 | rs17021918 | PDLIM5 | 4 | 94641726 | C | T | 0.323 |
| 63 | rs8102476 | SPINT2 | 19 | 38244973 | C | T | 0.622 |
| 64 | rs1571801 | DAB2IP | 9 | 121665094 | G | T | 0.067 |
| 65 | rs742134 | BIK | 22 | 43122269 | A | G | 0.867 |
| 66 | rs9375701 | L3MBTL3 | 6 | 130062912 | C | T | 0.713 |
| 67 | rs11649743 | HNF1B | 17 | 36074979 | G | A | 0.233 |
| 68 | rs7000448 | CASC8 | 8 | 127428925 | C | T | 0.252 |
| 69 | rs251177 | FCHSD1 | 5 | 141647317 | T | C | 0.133 |
| 70 | rs1465618 | THADA | 2 | 43326810 | T | C | 0.533 |

| | | | | | | | |
|---|---|---|---|---|---|---|---|
| 71 | rs9364554 | SLC22A3 | 6 | 160412632 | C | T | 0.270 |
| 72 | rs17181170 | AC107204.1 | 3 | 87124174 | G | A | 0.340 |
| 73 | rs1512268 | 8p21.2 | 8 | 23668950 | T | C | 0.588 |
| 74 | rs339331 | RFX6 | 6 | 116888889 | T | C | 0.392 |
| 75 | rs13252298 | AC020688.1 | 8 | 127082911 | A | G | 0.424 |
| 76 | rs103294 | AC245884.5 | 19 | 54797848 | C | T | 0.412 |
| 77 | rs4962416 | CTBP2 | 10 | 125008303 | T | C | 0.100 |
| 78 | rs11199874 | | 10 | 121273005 | G | A | 0.264 |
| 79 | rs137852593 | AR | X | 67717484 | G | T | 0.002 |
| 80 | rs2242652 | TERT | 5 | 1279913 | G | A | 0.211 |
| 81 | rs651164 | SLC22A1 | 6 | 160160342 | A | G | 0.479 |
| 82 | rs1469713 | GATAD2A | 19 | 19417997 | A | G | 0.080 |
| 83 | rs1529276 | | 13 | 103275657 | T | A | 0.078 |
| 84 | rs1983891 | FOXP4 | 6 | 41568689 | C | T | 0.308 |
| 85 | rs2107301 | VDR | 12 | 47861787 | G | A | 0.477 |
| 86 | rs445114 | CASC8 | 8 | 127310936 | T | C | 0.507 |
| 87 | rs10875943 | PRPH | 12 | 49282227 | T | C | 0.536 |
| 88 | rs16902094 | CASC8 | 8 | 127308101 | A | G | 0.252 |
| 89 | rs6763931 | ZBTB38 | 3 | 141383991 | G | A | 0.336 |
| 90 | rs9939609 | FTO | 16 | 53786615 | T | A | 0.310 |
| 91 | rs12543663 | PCAT1 | 8 | 126912414 | C | A | 0.758 |
| 92 | rs1859962 | CASC17 | 17 | 71112612 | G | T | 0.530 |
| 93 | rs6983561 | PRNCR1 | 8 | 127094635 | A | C | 0.102 |
| 94 | rs731236 | VDR | 12 | 48238757 | A | G | 0.232 |
| 95 | rs10486567 | JAZF1 | 7 | 27936944 | G | A | 0.478 |
| 96 | rs902774 | KRT8 | 12 | 53273904 | G | A | 0.079 |
| 97 | rs4792311 | ELAC2 | 17 | 13011692 | G | A | 0.209 |
| 98 | rs721048 | EHBP1 | 2 | 62904596 | G | A | 0.078 |
| 99 | rs2735839 | KLK3 | 19 | 51364623 | G | A | 0.302 |
| 100 | rs6738028 | AC108463.2 | 2 | 111191750 | G | C | 0.519 |
| 101 | rs12653946 | AC025183.2 | 5 | 1895715 | C | T | 0.474 |
| 102 | rs16901979 | CASC19 | 8 | 127112671 | C | A | 0.117 |

Table S2. **Genotype distributions in controls and cases (all 102 SNPs)**

| SNP | Alleles (M/m) | Ctrl MM | Ctrl Mm | Ctrl mm | Case MM | Case Mm | Case mm | Ctrl MAF | Case MAF |
|---|---|---|---|---|---|---|---|---|---|
| rs76934034 | T/C | 280 (90.0) | 29 (9.3) | 2 (0.6) | 365 (89.5) | 43 (10.5) | 0 (0.0) | 0.053 | 0.053 |
| rs10009409 | C/T | 106 (35.7) | 149 (50.2) | 42 (14.1) | 135 (33.6) | 199 (49.5) | 68 (16.9) | 0.392 | 0.417 |
| rs684232 | T/C | 113 (33.6) | 166 (49.4) | 57 (17.0) | 151 (35.5) | 195 (45.9) | 79 (18.6) | 0.417 | 0.415 |
| rs1016343 | C/T | 248 (67.4) | 116 (31.5) | 4 (1.1) | 258 (59.0) | 165 (37.8) | 14 (3.2) | 0.168 | 0.221 |
| rs74315365 | C/T | 347 (99.4) | 2 (0.6) | 0 (0.0) | 436 (99.8) | 1 (0.2) | 0 (0.0) | 0.003 | 0.001 |
| rs10492519 | A/G | 247 (65.0) | 114 (30.0) | 19 (5.0) | 232 (53.7) | 168 (38.9) | 32 (7.4) | 0.200 | 0.269 |
| rs13385191 | A/G | 132 (31.4) | 196 (46.7) | 92 (21.9) | 145 (31.7) | 221 (48.3) | 92 (20.1) | 0.452 | 0.442 |
| rs2987983 | A/G | 157 (48.0) | 136 (41.6) | 34 (10.4) | 203 (49.6) | 166 (40.6) | 40 (9.8) | 0.312 | 0.301 |
| rs12155172 | G/A | 207 (50.7) | 178 (43.6) | 23 (5.6) | 254 (56.3) | 166 (36.8) | 31 (6.9) | 0.275 | 0.253 |
| rs12827748 | C/T | 82 (34.0) | 116 (48.1) | 43 (17.8) | 93 (24.5) | 219 (57.6) | 68 (17.9) | 0.419 | 0.467 |
| rs10498792 | T/C | 159 (68.2) | 43 (18.5) | 31 (13.3) | 277 (72.5) | 97 (25.4) | 8 (2.1) | 0.225 | 0.148 |
| rs11650494 | G/A | 314 (88.0) | 42 (11.8) | 1 (0.3) | 377 (88.7) | 44 (10.4) | 4 (0.9) | 0.062 | 0.061 |
| rs6465657 | C/T | 159 (48.9) | 151 (46.5) | 15 (4.6) | 219 (52.0) | 154 (36.6) | 48 (11.4) | 0.278 | 0.297 |
| rs121908982 | G/A | 420 (99.5) | 2 (0.5) | 0 (0.0) | 459 (99.8) | 1 (0.2) | 0 (0.0) | 0.002 | 0.001 |
| rs12418451 | G/A | 237 (69.3) | 26 (7.6) | 79 (23.1) | 274 (64.0) | 95 (22.2) | 59 (13.8) | 0.269 | 0.249 |
| rs345013 | A/G | 199 (69.1) | 79 (27.4) | 10 (3.5) | 264 (66.5) | 125 (31.5) | 8 (2.0) | 0.172 | 0.178 |
| rs7652331 | C/T | 247 (63.5) | 125 (32.1) | 17 (4.4) | 255 (58.8) | 146 (33.6) | 33 (7.6) | 0.204 | 0.244 |
| rs10187424 | T/C | 114 (27.1) | 280 (66.5) | 27 (6.4) | 121 (26.7) | 85 (18.7) | 248 (54.6) | 0.397 | 0.640 |
| rs13149290 | T/C | 105 (28.0) | 182 (48.5) | 88 (23.5) | 107 (24.6) | 204 (46.9) | 124 (28.5) | 0.477 | 0.520 |
| rs3217869 | A/G | 168 (42.0) | 199 (49.8) | 33 (8.3) | 178 (40.3) | 199 (45.0) | 65 (14.7) | 0.331 | 0.372 |
| rs2660753 | C/T | 226 (55.9) | 151 (37.4) | 27 (6.7) | 283 (62.7) | 141 (31.3) | 27 (6.0) | 0.254 | 0.216 |
| rs17361950 | C/T | 301 (89.6) | 20 (6.0) | 15 (4.5) | 387 (92.1) | 33 (7.9) | 0 (0.0) | 0.074 | 0.039 |
| rs887391 | T/C | 192 (49.0) | 167 (42.6) | 33 (8.4) | 264 (59.5) | 157 (35.4) | 23 (5.2) | 0.297 | 0.229 |
| rs5919432 | T/C | 363 (91.0) | 5 (1.3) | 31 (7.8) | 386 (86.9) | 21 (4.7) | 37 (8.3) | 0.084 | 0.107 |
| rs6545977 | G/A | 123 (33.4) | 180 (48.9) | 65 (17.7) | 119 (27.4) | 198 (45.6) | 117 (27.0) | 0.421 | 0.498 |
| rs4775302 | A/G | 66 (24.2) | 143 (52.4) | 64 (23.4) | 96 (24.7) | 177 (45.5) | 116 (29.8) | 0.496 | 0.526 |

| | | | | | | | | | |
|---|---|---|---|---|---|---|---|---|---|
| rs2238135 | C/G | 201 (50.8) | 163 (41.2) | 32 (8.1) | 247 (55.1) | 174 (38.8) | 27 (6.0) | 0.287 | 0.254 |
| rs7679673 | A/C | 113 (39.4) | 133 (46.3) | 41 (14.3) | 133 (32.8) | 202 (49.8) | 71 (17.5) | 0.375 | 0.424 |
| rs817826 | T/C | 258 (86.0) | 42 (14.0) | 0 (0.0) | 328 (79.4) | 78 (18.9) | 7 (1.7) | 0.070 | 0.111 |
| rs9311171 | T/G | 305 (74.2) | 100 (24.3) | 6 (1.5) | 365 (80.2) | 79 (17.4) | 11 (2.4) | 0.136 | 0.111 |
| rs9600079 | G/T | 108 (33.0) | 146 (44.6) | 73 (22.3) | 116 (27.6) | 230 (54.8) | 74 (17.6) | 0.446 | 0.450 |
| rs1456315 | C/T | 75 (29.9) | 134 (53.4) | 42 (16.7) | 106 (34.5) | 141 (45.9) | 60 (19.5) | 0.434 | 0.425 |
| rs137853009 | C/T | 233 (66.8) | 116 (33.2) | 0 (0.0) | 312 (73.2) | 114 (26.8) | 0 (0.0) | 0.166 | 0.134 |
| rs17041869 | A/G | 200 (84.7) | 26 (11.0) | 10 (4.2) | 284 (75.9) | 76 (20.3) | 14 (3.7) | 0.097 | 0.139 |
| rs1447295 | C/A | 208 (71.2) | 82 (28.1) | 2 (0.7) | 311 (79.3) | 72 (18.4) | 9 (2.3) | 0.147 | 0.115 |
| rs7127900 | G/A | 176 (61.3) | 90 (31.4) | 21 (7.3) | 243 (61.4) | 132 (33.3) | 21 (5.3) | 0.230 | 0.220 |
| rs119484087 | T/A | 277 (99.3) | 2 (0.7) | 0 (0.0) | 391 (100.0) | 0 (0.0) | 0 (0.0) | 0.004 | 0.000 |
| rs7153648 | G/C | 297 (77.1) | 79 (20.5) | 9 (2.3) | 339 (78.3) | 87 (20.1) | 7 (1.6) | 0.126 | 0.117 |
| rs7501939 | C/T | 131 (40.2) | 105 (32.2) | 90 (27.6) | 187 (44.6) | 154 (36.8) | 78 (18.6) | 0.437 | 0.370 |
| rs10934853 | C/A | 176 (41.4) | 187 (44.0) | 62 (14.6) | 196 (42.4) | 201 (43.5) | 65 (14.1) | 0.366 | 0.358 |
| rs6983267 | G/T | 69 (29.1) | 112 (47.3) | 56 (23.6) | 85 (22.3) | 201 (52.6) | 96 (25.1) | 0.473 | 0.514 |
| rs629242 | C/T | 197 (55.0) | 127 (35.5) | 34 (9.5) | 251 (57.3) | 155 (35.4) | 32 (7.3) | 0.272 | 0.250 |
| rs17599629 | A/G | 49 (60.5) | 29 (35.8) | 3 (3.7) | 137 (58.8) | 81 (34.8) | 15 (6.4) | 0.216 | 0.238 |
| rs121909139 | A/G | 271 (69.7) | 118 (30.3) | 0 (0.0) | 333 (74.3) | 115 (25.7) | 0 (0.0) | 0.152 | 0.128 |
| rs4430796 | A/G | 126 (34.1) | 184 (49.9) | 59 (16.0) | 163 (37.4) | 202 (46.3) | 71 (16.3) | 0.409 | 0.394 |
| rs37181 | T/G | 89 (47.1) | 51 (27.0) | 49 (25.9) | 164 (46.5) | 158 (44.8) | 31 (8.8) | 0.394 | 0.312 |
| rs12500426 | A/C | 61 (24.1) | 139 (54.9) | 53 (20.9) | 108 (28.8) | 190 (50.7) | 77 (20.5) | 0.484 | 0.459 |
| rs121908701 | C/C | 312 (100.0) | 312 (100.0) | 312 (100.0) | 407 (99.8) | 407 (99.8) | 407 (99.8) | 1.000 | 0.999 |
| rs12621278 | A/G | 249 (80.6) | 23 (7.4) | 37 (12.0) | 342 (83.4) | 64 (15.6) | 4 (1.0) | 0.157 | 0.088 |
| rs4466137 | G/T | 121 (58.5) | 76 (36.7) | 10 (4.8) | 176 (49.0) | 157 (43.7) | 26 (7.2) | 0.232 | 0.291 |
| rs10993994 | G/A | 130 (33.2) | 225 (57.4) | 37 (9.4) | 143 (32.7) | 230 (52.6) | 64 (14.6) | 0.381 | 0.410 |
| rs11228565 | G/A | 334 (78.2) | 85 (19.9) | 8 (1.9) | 200 (45.5) | 186 (42.3) | 54 (12.3) | 0.118 | 0.334 |

| | | | | | | | | | |
|---|---|---|---|---|---|---|---|---|---|
| rs5945572 | G/A | 302 (71.9) | 8 (1.9) | 110 (26.2) | 312 (68.7) | 48 (10.6) | 94 (20.7) | 0.271 | 0.260 |
| rs10896449 | A/G | 152 (49.8) | 113 (37.0) | 40 (13.1) | 195 (47.3) | 173 (42.0) | 44 (10.7) | 0.316 | 0.317 |
| rs11214775 | G/A | 93 (42.7) | 100 (45.9) | 25 (11.5) | 185 (50.0) | 158 (42.7) | 27 (7.3) | 0.344 | 0.286 |
| rs4245739 | A/C | 167 (68.7) | 72 (29.6) | 4 (1.6) | 252 (66.1) | 117 (30.7) | 12 (3.1) | 0.165 | 0.185 |
| rs2292884 | A/G | 190 (48.6) | 160 (40.9) | 41 (10.5) | 220 (49.1) | 192 (42.9) | 36 (8.0) | 0.309 | 0.295 |
| rs1545985 | A/G | 138 (60.8) | 89 (39.2) | 0 (0.0) | 73 (18.8) | 211 (54.4) | 104 (26.8) | 0.196 | 0.540 |
| rs4054823 | C/T | 121 (31.2) | 183 (47.2) | 84 (21.6) | 138 (31.5) | 221 (50.5) | 79 (18.0) | 0.452 | 0.433 |
| rs2121875 | A/C | 57 (23.8) | 159 (66.5) | 23 (9.6) | 137 (36.2) | 203 (53.7) | 38 (10.1) | 0.429 | 0.369 |
| rs636291 | A/G | 104 (46.0) | 89 (39.4) | 33 (14.6) | 178 (46.7) | 159 (41.7) | 44 (11.5) | 0.343 | 0.324 |
| rs17021918 | C/T | 181 (45.8) | 173 (43.8) | 41 (10.4) | 243 (54.1) | 162 (36.1) | 44 (9.8) | 0.323 | 0.278 |
| rs8102476 | T/C | 158 (38.8) | 190 (46.7) | 59 (14.5) | 146 (32.7) | 238 (53.2) | 63 (14.1) | 0.378 | 0.407 |
| rs1571801 | G/T | 349 (86.6) | 54 (13.4) | 0 (0.0) | 343 (75.7) | 94 (20.8) | 16 (3.5) | 0.067 | 0.139 |
| rs742134 | G/A | 274 (75.3) | 83 (22.8) | 7 (1.9) | 332 (76.3) | 99 (22.8) | 4 (0.9) | 0.133 | 0.123 |
| rs9375701 | T/C | 77 (57.5) | 37 (27.6) | 20 (14.9) | 115 (41.7) | 128 (46.4) | 33 (12.0) | 0.287 | 0.351 |
| rs11649743 | G/A | 225 (58.1) | 144 (37.2) | 18 (4.7) | 263 (59.8) | 156 (35.5) | 21 (4.8) | 0.233 | 0.225 |
| rs7000448 | C/T | 222 (56.8) | 141 (36.1) | 28 (7.2) | 203 (46.0) | 174 (39.5) | 64 (14.5) | 0.252 | 0.342 |
| rs251177 | T/C | 315 (77.8) | 72 (17.8) | 18 (4.4) | 329 (73.8) | 109 (24.4) | 8 (1.8) | 0.133 | 0.140 |
| rs1465618 | C/T | 107 (25.1) | 241 (56.4) | 79 (18.5) | 137 (30.1) | 227 (49.9) | 91 (20.0) | 0.467 | 0.449 |
| rs9364554 | C/T | 148 (53.6) | 107 (38.8) | 21 (7.6) | 194 (50.5) | 151 (39.3) | 39 (10.2) | 0.270 | 0.298 |
| rs17181170 | G/A | 127 (43.2) | 134 (45.6) | 33 (11.2) | 151 (37.3) | 202 (49.9) | 52 (12.8) | 0.340 | 0.378 |
| rs1512268 | C/T | 124 (30.5) | 231 (56.8) | 52 (12.8) | 158 (35.4) | 207 (46.4) | 81 (18.2) | 0.412 | 0.414 |
| rs339331 | T/C | 152 (36.0) | 209 (49.5) | 61 (14.5) | 191 (41.7) | 215 (46.9) | 52 (11.4) | 0.392 | 0.348 |
| rs13252298 | A/G | 94 (31.6) | 154 (51.9) | 49 (16.5) | 179 (44.1) | 192 (47.3) | 35 (8.6) | 0.424 | 0.323 |
| rs103294 | C/T | 131 (36.5) | 160 (44.6) | 68 (18.9) | 165 (38.1) | 202 (46.7) | 66 (15.2) | 0.412 | 0.386 |
| rs4962416 | T/C | 200 (81.3) | 43 (17.5) | 3 (1.2) | 296 (76.9) | 78 (20.3) | 11 (2.9) | 0.100 | 0.130 |
| rs11199874 | G/A | 203 (52.1) | 168 (43.1) | 19 (4.9) | 234 (53.1) | 170 (38.5) | 37 (8.4) | 0.264 | 0.277 |

| | | | | | | | | | |
|---|---|---|---|---|---|---|---|---|---|
| rs137852593 | G/T | 258 (99.6) | 1 (0.4) | 0 (0.0) | 369 (100.0) | 0 (0.0) | 0 (0.0) | 0.002 | 0.000 |
| rs2242652 | G/A | 204 (61.1) | 119 (35.6) | 11 (3.3) | 271 (64.4) | 114 (27.1) | 36 (8.6) | 0.211 | 0.221 |
| rs651164 | A/G | 63 (21.7) | 176 (60.7) | 51 (17.6) | 70 (17.4) | 257 (63.8) | 76 (18.9) | 0.479 | 0.507 |
| rs1469713 | A/G | 316 (84.0) | 60 (16.0) | 0 (0.0) | 217 (50.2) | 213 (49.3) | 2 (0.5) | 0.080 | 0.251 |
| rs1529276 | T/A | 173 (84.4) | 32 (15.6) | 0 (0.0) | 269 (71.9) | 96 (25.7) | 9 (2.4) | 0.078 | 0.152 |
| rs1983891 | C/T | 190 (47.5) | 174 (43.5) | 36 (9.0) | 236 (53.0) | 170 (38.2) | 39 (8.8) | 0.307 | 0.279 |
| rs2107301 | G/A | 81 (25.2) | 175 (54.3) | 66 (20.5) | 136 (33.3) | 186 (45.5) | 87 (21.3) | 0.477 | 0.440 |
| rs445114 | C/T | 52 (17.5) | 197 (66.3) | 48 (16.2) | 40 (13.7) | 177 (60.6) | 75 (25.7) | 0.493 | 0.560 |
| rs10875943 | C/T | 121 (31.1) | 175 (45.0) | 93 (23.9) | 116 (26.1) | 236 (53.0) | 93 (20.9) | 0.464 | 0.474 |
| rs16902094 | A/G | 120 (53.6) | 95 (42.4) | 9 (4.0) | 211 (55.1) | 148 (38.6) | 24 (6.3) | 0.252 | 0.256 |
| rs6763931 | G/A | 169 (44.0) | 172 (44.8) | 43 (11.2) | 184 (41.8) | 182 (41.4) | 74 (16.8) | 0.336 | 0.375 |
| rs9939609 | T/A | 139 (47.9) | 122 (42.1) | 29 (10.0) | 215 (53.1) | 141 (34.8) | 49 (12.1) | 0.310 | 0.295 |
| rs12543663 | A/C | 205 (57.3) | 133 (37.2) | 20 (5.6) | 245 (57.0) | 147 (34.2) | 38 (8.8) | 0.242 | 0.259 |
| rs1859962 | T/G | 97 (26.6) | 193 (52.9) | 75 (20.5) | 103 (23.8) | 208 (48.1) | 121 (28.0) | 0.470 | 0.521 |
| rs6983561 | A/C | 169 (80.5) | 39 (18.6) | 2 (1.0) | 284 (76.5) | 83 (22.4) | 4 (1.1) | 0.102 | 0.123 |
| rs731236 | A/G | 216 (57.9) | 141 (37.8) | 16 (4.3) | 244 (55.8) | 171 (39.1) | 22 (5.0) | 0.232 | 0.246 |
| rs10486567 | G/A | 65 (28.6) | 107 (47.1) | 55 (24.2) | 86 (23.2) | 190 (51.4) | 94 (25.4) | 0.478 | 0.511 |
| rs902774 | G/A | 338 (85.1) | 55 (13.9) | 4 (1.0) | 361 (81.9) | 70 (15.9) | 10 (2.3) | 0.079 | 0.102 |
| rs4792311 | G/A | 253 (64.9) | 111 (28.5) | 26 (6.7) | 293 (65.7) | 130 (29.1) | 23 (5.2) | 0.209 | 0.197 |
| rs721048 | G/A | 270 (84.4) | 50 (15.6) | 0 (0.0) | 344 (81.3) | 79 (18.7) | 0 (0.0) | 0.078 | 0.093 |
| rs2735839 | G/A | 213 (49.7) | 173 (40.3) | 43 (10.0) | 276 (60.3) | 159 (34.7) | 23 (5.0) | 0.302 | 0.224 |
| rs6738028 | C/G | 72 (24.2) | 164 (55.2) | 61 (20.5) | 142 (34.7) | 187 (45.7) | 80 (19.6) | 0.481 | 0.424 |
| rs12653946 | C/T | 104 (26.7) | 202 (51.8) | 84 (21.5) | 119 (26.6) | 216 (48.3) | 112 (25.1) | 0.474 | 0.492 |
| rs16901979 | C/A | 143 (77.7) | 39 (21.2) | 2 (1.1) | 244 (70.9) | 95 (27.6) | 5 (1.5) | 0.117 | 0.153 |

Genotypes ordered major-allele homozygote (MM) / heterozygote (Mm) / minor-allele homozygote (mm); counts are n (%) of successfully genotyped participants per group. M = major allele, m = minor allele

Table S3. **Hardy–Weinberg equilibrium in the control group (all 102 SNPs)**

| SNP | Alleles (M/m) | MM | Mm | mm | Ctrl MAF | HWE p |
|---|---|---|---|---|---|---|
| rs76934034 | T/C | 280 | 29 | 2 | 0.053 | 0.204 |
| rs10009409 | C/T | 106 | 149 | 42 | 0.392 | 0.368 |
| rs684232 | T/C | 113 | 166 | 57 | 0.417 | 0.765 |
| rs1016343 | C/T | 248 | 116 | 4 | 0.168 | 0.016 |
| rs74315365 | C/T | 347 | 2 | 0 | 0.003 | 0.957 |
| rs10492519 | A/G | 247 | 114 | 19 | 0.200 | 0.223 |
| rs13385191 | A/G | 132 | 196 | 92 | 0.452 | 0.234 |
| rs2987983 | A/G | 157 | 136 | 34 | 0.312 | 0.574 |
| rs12155172 | G/A | 207 | 178 | 23 | 0.275 | 0.054 |
| rs12827748 | C/T | 82 | 116 | 43 | 0.419 | 0.859 |
| rs10498792 | T/C | 159 | 43 | 31 | 0.225 | <0.001 |
| rs11650494 | G/A | 314 | 42 | 1 | 0.062 | 0.745 |
| rs6465657 | C/T | 159 | 151 | 15 | 0.278 | 0.005 |
| rs121908982 | G/A | 420 | 2 | 0 | 0.002 | 0.961 |
| rs12418451 | G/A | 237 | 26 | 79 | 0.269 | <0.001 |
| rs345013 | A/G | 199 | 79 | 10 | 0.172 | 0.537 |
| rs7652331 | C/T | 247 | 125 | 17 | 0.204 | 0.814 |
| rs10187424 | T/C | 114 | 280 | 27 | 0.397 | <0.001 |
| rs13149290 | T/C | 105 | 182 | 88 | 0.477 | 0.597 |
| rs3217869 | A/G | 168 | 199 | 33 | 0.331 | 0.014 |
| rs2660753 | C/T | 226 | 151 | 27 | 0.254 | 0.794 |
| rs17361950 | C/T | 301 | 20 | 15 | 0.074 | <0.001 |
| rs887391 | T/C | 192 | 167 | 33 | 0.297 | 0.695 |
| rs5919432 | T/C | 363 | 5 | 31 | 0.084 | <0.001 |
| rs6545977 | G/A | 123 | 180 | 65 | 0.421 | 0.951 |
| rs4775302 | A/G | 66 | 143 | 64 | 0.496 | 0.431 |
| rs2238135 | C/G | 201 | 163 | 32 | 0.287 | 0.896 |
| rs7679673 | A/C | 113 | 133 | 41 | 0.375 | 0.853 |
| rs817826 | T/C | 258 | 42 | 0 | 0.070 | 0.192 |
| rs9311171 | T/G | 305 | 100 | 6 | 0.136 | 0.494 |
| rs9600079 | G/T | 108 | 146 | 73 | 0.446 | 0.080 |
| rs1456315 | C/T | 75 | 134 | 42 | 0.434 | 0.171 |
| rs137853009 | C/T | 233 | 116 | 0 | 0.166 | <0.001 |
| rs17041869 | A/G | 200 | 26 | 10 | 0.097 | <0.001 |
| rs1447295 | C/A | 208 | 82 | 2 | 0.147 | 0.043 |
| rs7127900 | G/A | 176 | 90 | 21 | 0.230 | 0.052 |
| rs119484087 | T/A | 277 | 2 | 0 | 0.004 | 0.952 |
| rs7153648 | G/C | 297 | 79 | 9 | 0.126 | 0.181 |
| rs7501939 | C/T | 131 | 105 | 90 | 0.437 | <0.001 |
| rs10934853 | C/A | 176 | 187 | 62 | 0.366 | 0.286 |
| rs6983267 | G/T | 69 | 112 | 56 | 0.473 | 0.423 |
| rs629242 | C/T | 197 | 127 | 34 | 0.272 | 0.047 |
| rs17599629 | A/G | 49 | 29 | 3 | 0.216 | 0.608 |
| rs121909139 | A/G | 271 | 118 | 0 | 0.152 | <0.001 |
| rs4430796 | A/G | 126 | 184 | 59 | 0.409 | 0.548 |
| rs37181 | T/G | 89 | 51 | 49 | 0.394 | <0.001 |
| rs12500426 | A/C | 61 | 139 | 53 | 0.484 | 0.112 |
| rs121908701 | C/C | 312 | 312 | 312 | 1.000 | <0.001 |
| rs12621278 | A/G | 249 | 23 | 37 | 0.157 | <0.001 |

| | | | | | | |
|---|---|---|---|---|---|---|
| rs4466137 | G/T | 121 | 76 | 10 | 0.232 | 0.659 |
| rs10993994 | G/A | 130 | 225 | 37 | 0.381 | <0.001 |
| rs11228565 | G/A | 334 | 85 | 8 | 0.118 | 0.347 |
| rs5945572 | G/A | 302 | 8 | 110 | 0.271 | <0.001 |
| rs10896449 | A/G | 152 | 113 | 40 | 0.316 | 0.012 |
| rs11214775 | G/A | 93 | 100 | 25 | 0.344 | 0.810 |
| rs4245739 | A/C | 167 | 72 | 4 | 0.165 | 0.228 |
| rs2292884 | A/G | 190 | 160 | 41 | 0.309 | 0.400 |
| rs1545985 | A/G | 138 | 89 | 0 | 0.196 | <0.001 |
| rs4054823 | C/T | 121 | 183 | 84 | 0.452 | 0.344 |
| rs2121875 | A/C | 57 | 159 | 23 | 0.429 | <0.001 |
| rs636291 | A/G | 104 | 89 | 33 | 0.343 | 0.058 |
| rs17021918 | C/T | 181 | 173 | 41 | 0.323 | 0.972 |
| rs8102476 | T/C | 158 | 190 | 59 | 0.378 | 0.878 |
| rs1571801 | G/T | 349 | 54 | 0 | 0.067 | 0.149 |
| rs742134 | G/A | 274 | 83 | 7 | 0.133 | 0.807 |
| rs9375701 | T/C | 77 | 37 | 20 | 0.287 | <0.001 |
| rs11649743 | G/A | 225 | 144 | 18 | 0.233 | 0.404 |
| rs7000448 | C/T | 222 | 141 | 28 | 0.252 | 0.393 |
| rs251177 | T/C | 315 | 72 | 18 | 0.133 | <0.001 |
| rs1465618 | C/T | 107 | 241 | 79 | 0.467 | 0.006 |
| rs9364554 | C/T | 148 | 107 | 21 | 0.270 | 0.786 |
| rs17181170 | G/A | 127 | 134 | 33 | 0.340 | 0.792 |
| rs1512268 | C/T | 124 | 231 | 52 | 0.412 | <0.001 |
| rs339331 | T/C | 152 | 209 | 61 | 0.392 | 0.425 |
| rs13252298 | A/G | 94 | 154 | 49 | 0.424 | 0.290 |
| rs103294 | C/T | 131 | 160 | 68 | 0.412 | 0.128 |
| rs4962416 | T/C | 200 | 43 | 3 | 0.100 | 0.691 |
| rs11199874 | G/A | 203 | 168 | 19 | 0.264 | 0.033 |
| rs137852593 | G/T | 258 | 1 | 0 | 0.002 | 0.975 |
| rs2242652 | G/A | 204 | 119 | 11 | 0.211 | 0.202 |
| rs651164 | A/G | 63 | 176 | 51 | 0.479 | <0.001 |
| rs1469713 | A/G | 316 | 60 | 0 | 0.080 | 0.093 |
| rs1529276 | T/A | 173 | 32 | 0 | 0.078 | 0.225 |
| rs1983891 | C/T | 190 | 174 | 36 | 0.307 | 0.669 |
| rs2107301 | G/A | 81 | 175 | 66 | 0.477 | 0.109 |
| rs445114 | C/T | 52 | 197 | 48 | 0.493 | <0.001 |
| rs10875943 | C/T | 121 | 175 | 93 | 0.464 | 0.059 |
| rs16902094 | A/G | 120 | 95 | 9 | 0.252 | 0.063 |
| rs6763931 | G/A | 169 | 172 | 43 | 0.336 | 0.939 |
| rs9939609 | T/A | 139 | 122 | 29 | 0.310 | 0.769 |
| rs12543663 | A/C | 205 | 133 | 20 | 0.242 | 0.795 |
| rs1859962 | T/G | 97 | 193 | 75 | 0.470 | 0.241 |
| rs6983561 | A/C | 169 | 39 | 2 | 0.102 | 0.880 |
| rs731236 | A/G | 216 | 141 | 16 | 0.232 | 0.238 |
| rs10486567 | G/A | 65 | 107 | 55 | 0.478 | 0.404 |
| rs902774 | G/A | 338 | 55 | 4 | 0.079 | 0.303 |
| rs4792311 | G/A | 253 | 111 | 26 | 0.209 | 0.006 |
| rs721048 | G/A | 270 | 50 | 0 | 0.078 | 0.130 |
| rs2735839 | G/A | 213 | 173 | 43 | 0.302 | 0.371 |
| rs6738028 | C/G | 72 | 164 | 61 | 0.481 | 0.068 |
| rs12653946 | C/T | 104 | 202 | 84 | 0.474 | 0.446 |

| | | | | | | |
|---|---|---|---|---|---|---|
| rs16901979 | C/A | 143 | 39 | 2 | 0.117 | 0.714 |

Hardy–Weinberg equilibrium computed in controls only ($\chi^2$, 1 df). Of the 102 SNPs, 30 deviated from HWE in controls ($p < 0.05$). Among the 14 loci significant after correction, 8 also deviated — rs10187424, rs12621278, rs37181, rs10498792, rs1545985, rs17361950, rs6465657 and rs12418451 — and their associations are interpreted with particular caution, since HWE departure in controls can reflect genotyping error (main text, Section 3.2)

Table S4. **Genotypic associations for SNPs significant by genotype distribution ($\chi^2$, p < 0.05)**

| SNP (gene) | Genotype | Control n (%) | Case n (%) | $\chi^2$ | p |
|---|---|---|---|---|---|
| **rs10187424** *(VAMP8)* | TT | 114 (27.1) | 121 (26.7) | 281.15 | <0.0001 |
| | CT | 280 (66.5) | 85 (18.7) | | |
| | CC | 27 (6.4) | 248 (54.6) | | |
| **rs1545985** *(FYCO1)* | AA | 138 (60.8) | 73 (18.8) | 141.16 | <0.0001 |
| | AG | 89 (39.2) | 211 (54.4) | | |
| | GG | 0 (0.0) | 104 (26.8) | | |
| **rs11228565** *(11q13.3)* | GG | 334 (78.2) | 200 (45.5) | 105.23 | <0.0001 |
| | AG | 85 (19.9) | 186 (42.3) | | |
| | AA | 8 (1.9) | 54 (12.3) | | |
| **rs1469713** *(GATAD2A)* | AA | 316 (84.0) | 217 (50.2) | 102.75 | <0.0001 |
| | AG | 60 (16.0) | 213 (49.3) | | |
| | GG | 0 (0.0) | 2 (0.5) | | |
| **rs12621278** *(ITGA6)* | AA | 249 (80.6) | 342 (83.4) | 47.26 | <0.0001 |
| | AG | 23 (7.4) | 64 (15.6) | | |
| | GG | 37 (12.0) | 4 (1.0) | | |
| **rs12418451** *(AP003071.2)* | GG | 237 (69.3) | 274 (64.0) | 35.77 | <0.0001 |
| | AG | 26 (7.6) | 95 (22.2) | | |
| | AA | 79 (23.1) | 59 (13.8) | | |
| **rs37181** *(COMMD10)* | TT | 89 (47.1) | 164 (46.5) | 34.61 | <0.0001 |
| | GT | 51 (27.0) | 158 (44.8) | | |
| | GG | 49 (25.9) | 31 (8.8) | | |
| **rs10498792** *(PKHD1)* | TT | 159 (68.2) | 277 (72.5) | 32.11 | <0.0001 |
| | CT | 43 (18.5) | 97 (25.4) | | |
| | CC | 31 (13.3) | 8 (2.1) | | |
| **rs5945572** *(NUDT11)* | GG | 302 (71.9) | 312 (68.7) | 28.71 | <0.0001 |
| | AG | 8 (1.9) | 48 (10.6) | | |
| | AA | 110 (26.2) | 94 (20.7) | | |
| **rs1571801** *(DAB2IP)* | GG | 349 (86.6) | 343 (75.7) | 24.02 | <0.0001 |
| | GT | 54 (13.4) | 94 (20.8) | | |
| | TT | 0 (0.0) | 16 (3.5) | | |

| | | | | | |
|---|---|---|---|---|---|
| **rs17361950** *(FAAH)* | CC | 301 (89.6) | 387 (92.1) | 19.85 | <0.0001 |
| | CT | 20 (6.0) | 33 (7.9) | | |
| | TT | 15 (4.5) | 0 (0.0) | | |
| **rs13252298** *(AC020688.1)* | AA | 94 (31.6) | 179 (44.1) | 16.47 | 0.0003 |
| | AG | 154 (51.9) | 192 (47.3) | | |
| | GG | 49 (16.5) | 35 (8.6) | | |
| **rs7000448** *(CASC8)* | CC | 222 (56.8) | 203 (46.0) | 15.44 | 0.0004 |
| | CT | 141 (36.1) | 174 (39.5) | | |
| | TT | 28 (7.2) | 64 (14.5) | | |
| **rs6465657** *(LMTK2)* | CC | 159 (48.9) | 219 (52.0) | 14.73 | 0.0006 |
| | CT | 151 (46.5) | 154 (36.6) | | |
| | TT | 15 (4.6) | 48 (11.4) | | |
| **rs2735839** *(KLK3)* | GG | 213 (49.7) | 276 (60.3) | 13.83 | 0.0010 |
| | AG | 173 (40.3) | 159 (34.7) | | |
| | AA | 43 (10.0) | 23 (5.0) | | |
| **rs1529276** *(—)* | TT | 173 (84.4) | 269 (71.9) | 13.69 | 0.0011 |
| | AT | 32 (15.6) | 96 (25.7) | | |
| | AA | 0 (0.0) | 9 (2.4) | | |
| **rs9375701** *(L3MBTL3)* | TT | 77 (57.5) | 115 (41.7) | 13.31 | 0.0013 |
| | CT | 37 (27.6) | 128 (46.4) | | |
| | CC | 20 (14.9) | 33 (12.0) | | |
| **rs2242652** *(TERT)* | GG | 204 (61.1) | 271 (64.4) | 13.00 | 0.0015 |
| | AG | 119 (35.6) | 114 (27.1) | | |
| | AA | 11 (3.3) | 36 (8.6) | | |
| **rs2121875** *(FGF10)* | AA | 57 (23.8) | 137 (36.2) | 11.28 | 0.0035 |
| | AC | 159 (66.5) | 203 (53.7) | | |
| | CC | 23 (9.6) | 38 (10.1) | | |
| **rs1447295** *(CASC8)* | CC | 208 (71.2) | 311 (79.3) | 11.16 | 0.0038 |
| | AC | 82 (28.1) | 72 (18.4) | | |
| | AA | 2 (0.7) | 9 (2.3) | | |
| **rs10492519** *(FAM124A)* | AA | 247 (65.0) | 232 (53.7) | 10.84 | 0.0044 |
| | AG | 114 (30.0) | 168 (38.9) | | |
| | GG | 19 (5.0) | 32 (7.4) | | |
| **rs6545977** *(OTX1)* | GG | 123 (33.4) | 119 (27.4) | 10.42 | 0.0055 |
| | AG | 180 (48.9) | 198 (45.6) | | |
| | AA | 65 (17.7) | 117 (27.0) | | |
| **rs887391** *(PCAT19)* | TT | 192 (49.0) | 264 (59.5) | 10.27 | 0.0059 |
| | CT | 167 (42.6) | 157 (35.4) | | |
| | CC | 33 (8.4) | 23 (5.2) | | |
| **rs1512268** *(8p21.2)* | CC | 124 (30.5) | 158 (35.4) | 9.98 | 0.0068 |

| | | | | | |
|---|---|---|---|---|---|
| | CT | 231 (56.8) | 207 (46.4) | | |
| | TT | 52 (12.8) | 81 (18.2) | | |
| **rs251177** *(FCHSD1)* | TT | 315 (77.8) | 329 (73.8) | 9.76 | 0.0076 |
| | CT | 72 (17.8) | 109 (24.4) | | |
| | CC | 18 (4.4) | 8 (1.8) | | |
| **rs6738028** *(AC108463.2)* | CC | 72 (24.2) | 142 (34.7) | 9.43 | 0.0089 |
| | CG | 164 (55.2) | 187 (45.7) | | |
| | GG | 61 (20.5) | 80 (19.6) | | |
| **rs17041869** *(BCL2L11)* | AA | 200 (84.7) | 284 (75.9) | 9.00 | 0.0111 |
| | AG | 26 (11.0) | 76 (20.3) | | |
| | GG | 10 (4.2) | 14 (3.7) | | |
| **rs5919432** *(AR)* | TT | 363 (91.0) | 386 (86.9) | 8.70 | 0.0129 |
| | CT | 5 (1.3) | 21 (4.7) | | |
| | CC | 31 (7.8) | 37 (8.3) | | |
| **rs3217869** *(CCND2)* | AA | 168 (42.0) | 178 (40.3) | 8.66 | 0.0131 |
| | AG | 199 (49.8) | 199 (45.0) | | |
| | GG | 33 (8.3) | 65 (14.7) | | |
| **rs445114** *(CASC8)* | CC | 52 (17.5) | 40 (13.7) | 8.52 | 0.0141 |
| | CT | 197 (66.3) | 177 (60.6) | | |
| | TT | 48 (16.2) | 75 (25.7) | | |
| **rs7501939** *(HNF1B)* | CC | 131 (40.2) | 187 (44.6) | 8.51 | 0.0142 |
| | CT | 105 (32.2) | 154 (36.8) | | |
| | TT | 90 (27.6) | 78 (18.6) | | |
| **rs817826** *(KLF4)* | TT | 258 (86.0) | 328 (79.4) | 8.47 | 0.0145 |
| | CT | 42 (14.0) | 78 (18.9) | | |
| | CC | 0 (0.0) | 7 (1.7) | | |
| **rs1016343** *(CASC19)* | CC | 248 (67.4) | 258 (59.0) | 8.45 | 0.0147 |
| | CT | 116 (31.5) | 165 (37.8) | | |
| | TT | 4 (1.1) | 14 (3.2) | | |
| **rs9600079** *(KLF5)* | GG | 108 (33.0) | 116 (27.6) | 7.60 | 0.0224 |
| | GT | 146 (44.6) | 230 (54.8) | | |
| | TT | 73 (22.3) | 74 (17.6) | | |
| **rs12827748** *(PAWR)* | CC | 82 (34.0) | 93 (24.5) | 7.24 | 0.0268 |
| | CT | 116 (48.1) | 219 (57.6) | | |
| | TT | 43 (17.8) | 68 (17.9) | | |
| **rs9311171** *(CTDSPL)* | TT | 305 (74.2) | 365 (80.2) | 7.09 | 0.0289 |
| | GT | 100 (24.3) | 79 (17.4) | | |
| | GG | 6 (1.5) | 11 (2.4) | | |
| **rs2107301** *(VDR)* | GG | 81 (25.2) | 136 (33.3) | 6.90 | 0.0317 |
| | AG | 175 (54.3) | 186 (45.5) | | |
| | AA | 66 (20.5) | 87 (21.3) | | |

| | | | | | |
|---|---|---|---|---|---|
| **rs17021918** *(PDLIM5)* | CC | 181 (45.8) | 243 (54.1) | 6.10 | 0.0473 |
| | CT | 173 (43.8) | 162 (36.1) | | |
| | TT | 41 (10.4) | 44 (9.8) | | |
| **rs137853009** *(CHEK2)* | CC | 233 (66.8) | 312 (73.2) | 3.86 | 0.0496 |
| | CT | 116 (33.2) | 114 (26.8) | | |
| | TT | 0 (0.0) | 0 (0.0) | | |

Genotypes ordered major-homozygote / heterozygote / minor-homozygote. 39 SNPs reached genotypic $p < 0.05$; $\chi^2$ is the 2×k genotype test. Complete-separation loci with a zero genotype cell (rs1545985, rs1571801, rs17361950, rs1469713) yield unstable estimates and are interpreted with caution

Table S5. **Allelic associations for all 102 SNPs (minor-allele odds ratios)**

| SNP (gene) | Minor | Case m/M | Ctrl m/M | Allelic OR (95% CI) | p | Sig. |
|---|---|---|---|---|---|---|
| **rs1545985** *(FYCO1)* | G | 419/357 | 89/365 | 4.81 (3.67–6.31) | <0.0001 | **Bonf.** |
| **rs11228565** *(11q13.3)* | A | 294/586 | 101/753 | 3.74 (2.91–4.81) | <0.0001 | **Bonf.** |
| **rs10187424** *(VAMP8)* | C | 581/327 | 334/508 | 2.70 (2.23–3.28) | <0.0001 | **Bonf.** |
| **rs1469713** *(GATAD2A)* | G | 217/647 | 60/692 | 3.87 (2.85–5.25) | <0.0001 | **Bonf.** |
| **rs1571801** *(DAB2IP)* | T | 126/780 | 54/752 | 2.25 (1.61–3.14) | <0.0001 | **Bonf.** |
| **rs12621278** *(ITGA6)* | G | 72/748 | 97/521 | 0.52 (0.37–0.72) | <0.0001 | **Bonf.** |
| **rs7000448** *(CASC8)* | T | 302/580 | 197/585 | 1.55 (1.25–1.91) | <0.0001 | **Bonf.** |
| **rs13252298** *(AC020688.1)* | G | 262/550 | 252/342 | 0.65 (0.52–0.81) | <0.0001 | **Bonf.** |
| **rs2735839** *(KLK3)* | A | 205/711 | 259/599 | 0.67 (0.54–0.83) | 0.0002 | **Bonf.** |
| **rs1529276** *(—)* | A | 114/634 | 32/378 | 2.12 (1.41–3.21) | 0.0003 | **Bonf.** |
| **rs10498792** *(PKHD1)* | C | 113/651 | 105/361 | 0.60 (0.44–0.80) | 0.0006 | p<0.05 |
| **rs10492519** *(FAM124A)* | G | 232/632 | 152/608 | 1.47 (1.16–1.85) | 0.0012 | p<0.05 |
| **rs887391** *(PCAT19)* | C | 203/685 | 233/551 | 0.70 (0.56–0.87) | 0.0014 | p<0.05 |
| **rs6545977** *(OTX1)* | A | 432/436 | 310/426 | 1.36 (1.12–1.66) | 0.0022 | p<0.05 |
| **rs17361950** *(FAAH)* | T | 33/807 | 50/622 | 0.51 (0.32–0.80) | 0.0029 | p<0.05 |
| **rs37181** *(COMMD10)* | G | 220/486 | 149/229 | 0.70 (0.54–0.90) | 0.0063 | p<0.05 |
| **rs817826** *(KLF4)* | C | 92/734 | 42/558 | 1.67 (1.14–2.44) | 0.0082 | p<0.05 |
| **rs1016343** *(CASC19)* | T | 193/681 | 124/612 | 1.40 (1.09–1.80) | 0.0085 | p<0.05 |
| **rs7501939** *(HNF1B)* | T | 310/528 | 285/367 | 0.76 (0.61–0.93) | 0.0086 | p<0.05 |
| **rs445114** *(CASC8)* | T | 327/257 | 293/301 | 1.31 (1.04–1.64) | 0.0220 | p<0.05 |
| **rs4466137** *(HAPLN1)* | T | 209/509 | 96/318 | 1.36 (1.03–1.80) | 0.0306 | p<0.05 |
| **rs17041869** *(BCL2L11)* | G | 104/644 | 46/426 | 1.50 (1.04–2.16) | 0.0312 | p<0.05 |
| **rs6738028** *(AC108463.2)* | G | 347/471 | 286/308 | 0.79 (0.64–0.98) | 0.0326 | p<0.05 |
| **rs2121875** *(FGF10)* | C | 279/477 | 205/273 | 0.78 (0.62–0.98) | 0.0360 | p<0.05 |
| **rs11214775** *(HTR3B)* | A | 212/528 | 150/286 | 0.77 (0.59–0.99) | 0.0389 | p<0.05 |
| **rs1859962** *(CASC17)* | G | 450/414 | 343/387 | 1.23 (1.01–1.49) | 0.0426 | p<0.05 |
| **rs17021918** *(PDLIM5)* | T | 250/648 | 255/535 | 0.81 (0.66–1.00) | 0.0469 | p<0.05 |
| **rs7652331** *(FYCO1)* | T | 212/656 | 159/619 | 1.26 (1.00–1.59) | 0.0533 | ns |
| **rs339331** *(RFX6)* | C | 319/597 | 331/513 | 0.83 (0.68–1.01) | 0.0564 | ns |
| **rs7679673** *(AC004069.1)* | C | 344/468 | 215/359 | 1.23 (0.99–1.53) | 0.0665 | ns |
| **rs9375701** *(L3MBTL3)* | C | 194/358 | 77/191 | 1.34 (0.98–1.85) | 0.0670 | ns |
| **rs2660753** *(3p12)* | T | 195/707 | 205/603 | 0.81 (0.65–1.02) | 0.0672 | ns |
| **rs137853009** *(CHEK2)* | T | 114/738 | 116/582 | 0.78 (0.59–1.03) | 0.0743 | ns |
| **rs1447295** *(CASC8)* | A | 90/694 | 86/498 | 0.75 (0.55–1.03) | 0.0761 | ns |

| | | | | | | |
|---|---|---|---|---|---|---|
| **rs3217869** *(CCND2)* | G | 329/555 | 265/535 | 1.20 (0.98–1.46) | 0.0792 | ns |
| **rs13149290** *(ZNF827)* | C | 452/418 | 358/392 | 1.18 (0.97–1.44) | 0.0902 | ns |
| **rs119484087** *(ELAC2)* | A | 0/782 | 2/556 | 0.14 (0.01–2.97) † | 0.0939 | ns |
| **rs12827748** *(PAWR)* | T | 355/405 | 202/280 | 1.22 (0.97–1.53) | 0.0973 | ns |
| **rs6763931** *(ZBTB38)* | A | 330/550 | 258/510 | 1.19 (0.97–1.45) | 0.0987 | ns |
| **rs4962416** *(CTBP2)* | C | 100/670 | 49/443 | 1.35 (0.94–1.94) | 0.1040 | ns |
| **rs902774** *(KRT8)* | A | 90/792 | 63/731 | 1.32 (0.94–1.85) | 0.1072 | ns |
| **rs5919432** *(AR)* | C | 95/793 | 67/731 | 1.31 (0.94–1.82) | 0.1093 | ns |
| **rs9311171** *(CTDSPL)* | G | 101/809 | 112/710 | 0.79 (0.59–1.05) | 0.1099 | ns |
| **rs16901979** *(CASC19)* | A | 105/583 | 43/325 | 1.36 (0.93–1.99) | 0.1106 | ns |
| **rs2238135** *(AC121338.1)* | G | 228/668 | 227/565 | 0.85 (0.69–1.05) | 0.1374 | ns |
| **rs17181170** *(AC107204.1)* | A | 306/504 | 200/388 | 1.18 (0.94–1.47) | 0.1482 | ns |
| **rs6983267** *(CASC8)* | T | 393/371 | 224/250 | 1.18 (0.94–1.49) | 0.1525 | ns |
| **rs2107301** *(VDR)* | A | 360/458 | 307/337 | 0.86 (0.70–1.06) | 0.1629 | ns |
| **rs121909139** *(KLF6)* | G | 115/781 | 118/660 | 0.82 (0.62–1.09) | 0.1691 | ns |
| **rs1983891** *(FOXP4)* | T | 248/642 | 246/554 | 0.87 (0.71–1.07) | 0.1930 | ns |
| **rs8102476** *(SPINT2)* | C | 364/530 | 308/506 | 1.13 (0.93–1.37) | 0.2240 | ns |
| **rs137852593** *(AR)* | T | 0/738 | 1/517 | 0.23 (0.01–5.75) † | 0.2324 | ns |
| **rs10993994** *(MSMB)* | A | 358/516 | 299/485 | 1.13 (0.92–1.37) | 0.2406 | ns |
| **rs9364554** *(SLC22A3)* | T | 229/539 | 149/403 | 1.15 (0.90–1.47) | 0.2628 | ns |
| **rs10486567** *(JAZF1)* | A | 378/362 | 217/237 | 1.14 (0.90–1.44) | 0.2706 | ns |
| **rs103294** *(AC245884.5)* | T | 334/532 | 296/422 | 0.90 (0.73–1.10) | 0.2820 | ns |
| **rs4775302** *(AC091074.1)* | G | 409/369 | 271/275 | 1.12 (0.90–1.40) | 0.2926 | ns |
| **rs6983561** *(PRNCR1)* | C | 91/651 | 43/377 | 1.23 (0.83–1.80) | 0.2989 | ns |
| **rs721048** *(EHBP1)* | A | 79/767 | 50/590 | 1.22 (0.84–1.76) | 0.3010 | ns |
| **rs651164** *(SLC22A1)* | G | 409/397 | 278/302 | 1.12 (0.90–1.39) | 0.3014 | ns |
| **rs12155172** *(LINC01162)* | A | 228/674 | 224/592 | 0.89 (0.72–1.11) | 0.3068 | ns |
| **rs629242** *(KIAA1211)* | T | 219/657 | 195/521 | 0.89 (0.71–1.11) | 0.3119 | ns |
| **rs4245739** *(MDM4)* | C | 141/621 | 80/406 | 1.15 (0.85–1.56) | 0.3566 | ns |
| **rs10009409** *(—)* | T | 335/469 | 233/361 | 1.11 (0.89–1.37) | 0.3583 | ns |
| **rs12418451** *(AP003071.2)* | A | 213/643 | 184/500 | 0.90 (0.72–1.13) | 0.3685 | ns |
| **rs12500426** *(PDLIM5)* | C | 344/406 | 245/261 | 0.90 (0.72–1.13) | 0.3740 | ns |
| **rs12543663** *(PCAT1)* | C | 223/637 | 173/543 | 1.10 (0.87–1.38) | 0.4204 | ns |
| **rs4054823** *(—)* | T | 379/497 | 351/425 | 0.92 (0.76–1.12) | 0.4217 | ns |
| **rs6465657** *(LMTK2)* | T | 250/592 | 181/469 | 1.09 (0.87–1.37) | 0.4356 | ns |
| **rs74315365** *(RNASEL)* | T | 1/873 | 2/696 | 0.40 (0.04–4.41) | 0.4372 | ns |
| **rs1465618** *(THADA)* | T | 409/501 | 399/455 | 0.93 (0.77–1.12) | 0.4543 | ns |
| **rs12653946** *(AC025183.2)* | T | 440/454 | 370/410 | 1.07 (0.89–1.30) | 0.4670 | ns |
| **rs636291** *(PEX14)* | G | 247/515 | 155/297 | 0.92 (0.72–1.18) | 0.5016 | ns |
| **rs731236** *(VDR)* | G | 215/659 | 173/573 | 1.08 (0.86–1.36) | 0.5077 | ns |
| **rs2292884** *(MLPH)* | G | 264/632 | 242/540 | 0.93 (0.76–1.15) | 0.5093 | ns |
| **rs121908982** *(MAD1L1)* | A | 1/919 | 2/842 | 0.46 (0.04–5.06) | 0.5137 | ns |
| **rs9939609** *(FTO)* | A | 239/571 | 180/400 | 0.93 (0.74–1.17) | 0.5403 | ns |
| **rs742134** *(BIK)* | A | 107/763 | 97/631 | 0.91 (0.68–1.22) | 0.5407 | ns |
| **rs4430796** *(HNF1B)* | G | 344/528 | 302/436 | 0.94 (0.77–1.15) | 0.5483 | ns |
| **rs4792311** *(ELAC2)* | A | 176/716 | 163/617 | 0.93 (0.73–1.18) | 0.5539 | ns |
| **rs7153648** *(SIX1)* | C | 101/765 | 97/673 | 0.92 (0.68–1.23) | 0.5629 | ns |
| **rs17599629** *(GOLPH3L)* | G | 111/355 | 35/127 | 1.13 (0.74–1.75) | 0.5654 | ns |

| | | | | | | |
|---|---|---|---|---|---|---|
| **rs11199874** *(—)* | A | 244/638 | 206/574 | 1.07 (0.86–1.32) | 0.5658 | ns |
| **rs5945572** *(NUDT11)* | A | 236/672 | 228/612 | 0.94 (0.76–1.17) | 0.5859 | ns |
| **rs2987983** *(ESR2)* | G | 246/572 | 204/450 | 0.95 (0.76–1.19) | 0.6432 | ns |
| **rs2242652** *(TERT)* | A | 186/656 | 141/527 | 1.06 (0.83–1.36) | 0.6453 | ns |
| **rs7127900** *(TH)* | A | 174/618 | 132/442 | 0.94 (0.73–1.22) | 0.6532 | ns |
| **rs13385191** *(LDAH)* | G | 405/511 | 380/460 | 0.96 (0.79–1.16) | 0.6663 | ns |
| **rs10875943** *(PRPH)* | T | 422/468 | 361/417 | 1.04 (0.86–1.26) | 0.6787 | ns |
| **rs251177** *(FCHSD1)* | C | 125/767 | 108/702 | 1.06 (0.80–1.40) | 0.6835 | ns |
| **rs11649743** *(HNF1B)* | A | 198/682 | 180/594 | 0.96 (0.76–1.21) | 0.7149 | ns |
| **rs10934853** *(EEFSEC)* | A | 331/593 | 311/539 | 0.97 (0.80–1.17) | 0.7374 | ns |
| **rs1456315** *(AC020688.1)* | T | 261/353 | 218/284 | 0.96 (0.76–1.22) | 0.7579 | ns |
| **rs345013** *(—)* | G | 141/653 | 99/477 | 1.04 (0.78–1.38) | 0.7838 | ns |
| **rs16902094** *(CASC8)* | G | 196/570 | 113/335 | 1.02 (0.78–1.33) | 0.8882 | ns |
| **rs9600079** *(KLF5)* | T | 378/462 | 292/362 | 1.01 (0.83–1.25) | 0.8921 | ns |
| **rs1512268** *(8p21.2)* | T | 369/523 | 335/479 | 1.01 (0.83–1.22) | 0.9289 | ns |
| **rs684232** *(VPS53)* | C | 353/497 | 280/392 | 0.99 (0.81–1.22) | 0.9570 | ns |
| **rs11650494** *(AC004797.1)* | A | 52/798 | 44/670 | 0.99 (0.66–1.50) | 0.9707 | ns |
| **rs76934034** *(—)* | C | 43/773 | 33/589 | 0.99 (0.62–1.58) | 0.9760 | ns |
| **rs10896449** *(MYEOV)* | G | 261/563 | 193/417 | 1.00 (0.80–1.25) | 0.9886 | ns |
| **rs121908701** *(CHEK2)* | C | 815/815 | 624/624 | 1.00 (0.86–1.16) | 1.0000 | ns |

m/M = minor/major allele counts. OR is for the minor allele versus the major allele. "Bonf." = significant at the Bonferroni threshold ($p < 0.05/102 = 0.00049$); "p<0.05" = nominal. OR obtained with the Haldane–Anscombe correction (a zero allele cell).

Table S6. **Age-adjusted associations for the 14 significant SNPs (sensitivity analysis)**

| SNP (gene) | HWE-consistent (controls) | Crude OR | Age-adjusted OR (95% CI) | p (adj) | Verdict |
|---|---|---|---|---|---|
| **rs10187424** *(VAMP8)* | no | 2.76 | 2.57 (2.05–3.23) | **<0.0001** | **significant** |
| **rs1545985** *(FYCO1)* | no | 7.08 | 6.73 (4.58–9.91) | **<0.0001** | **significant** |
| **rs11228565** *(11q13.3)* | **yes** | 4.02 | 4.06 (3.02–5.45) | **<0.0001** | **significant** |
| **rs12621278** *(ITGA6)* | no | 0.61 | 0.62 (0.45–0.86) | **0.0045** | **significant** |
| **rs37181** *(COMMD10)* | no | 0.70 | 0.71 (0.54–0.93) | **0.0127** | **significant** |
| **rs10498792** *(PKHD1)* | no | 0.66 | 0.76 (0.56–1.04) | 0.0821 | ns |
| **rs1571801** *(DAB2IP)* | **yes** | 2.02 | 2.02 (1.38–2.94) | **0.0003** | **significant** |
| **rs17361950** *(FAAH)* | no | 0.59 | 0.62 (0.38–1.01) | 0.0550 | ns |
| **rs7000448** *(CASC8)* | **yes** | 1.55 | 1.62 (1.27–2.08) | **0.0001** | **significant** |
| **rs13252298** *(AC020688.1)* | **yes** | 0.64 | 0.66 (0.51–0.86) | **0.0021** | **significant** |
| **rs6465657** *(LMTK2)* | no | 1.03 | 1.02 (0.78–1.33) | 0.8935 | ns |
| **rs887391** *(PCAT19)* | **yes** | 0.64 | 0.56 (0.43–0.73) | **<0.0001** | **significant** |
| **rs1469713** *(GATAD2A)* | **yes** | 5.29 | 5.02 (3.45–7.31) | **<0.0001** | **significant** |
| **rs12418451** *(HNF1B)* | no | 0.96 | 0.95 (0.77–1.18) | 0.6650 | ns |

Additive (minor-allele dosage) logistic regression with age as a covariate, in the 794 participants with a recorded age (cases mean 62.5 y, controls 52.9 y). 10 of the 14 loci remained significant after age adjustment. Six loci were BOTH significant after age adjustment AND in Hardy–Weinberg equilibrium in controls - rs11228565, rs1571801, rs7000448, rs13252298, rs887391 and rs1469713 - and represent a core association signal robust to both the age imbalance and control-group HWE concerns. rs10498792 and rs17361950

became borderline after adjustment; rs6465657 and rs12418451 were not significant under the additive model

Table S7. **Risk classes identified using the decision tree method**

| № | Class definition | Group size | Class share. % | Risk. % |
|---|---|---|---|---|
| **1** | **2** | **3** | **4** | **5** |
| 1 | Rs10187424 (C/C) & Rs1469713 (A/G) | 126 | 17.4 | 100.0 |
| 2 | Rs10187424 (C/C) & Rs1469713 (A/A) & Rs5945572 (G/G. A/G) | 97 | 13.4 | 89.7 |
| 3 | Rs10187424 (C/C) & Rs1469713 (A/A) & Rs5945572 (A/A) | 23 | 3.2 | 73.9 |
| 4 | Rs10187424 (C/T. T/T) & Rs1469713 (A/G) & Rs9600079 (G/T) | 77 | 10.7 | 64.9 |
| 5 | Rs10187424 (C/T. T/T) & Rs1469713 (A/A. G/G) & Rs887391 (T/T) & Rs9600079 (G/T) | 81 | 11.2 | 48.1 |
| 6 | Rs10187424 (C/T. T/T) & Rs1469713 (A/G) & Rs9600079 (T/T. G/G) | 63 | 8.7 | 47.6 |
| 7 | Rs10187424 (C/T. T/T) & Rs1469713 (A/A. G/G) & Rs887391 (C/T. C/C) & Rs6465657 (C/C. T/T) | 99 | 13.7 | 29.3 |
| 8 | Rs10187424 (C/T. T/T) & Rs1469713 (A/A. G/G) & Rs887391 (T/T) & Rs9600079 (G/G. T/T) | 94 | 13.0 | 27.7 |
| 9 | Rs10187424 (C/T. T/T) & Rs1469713 (A/A. G/G) & Rs887391 (C/T. C/C) & Rs6465657 (C/T) | 63 | 8.7 | 9.5 |

Terminal classes of the CART decision tree. The X-linked rs5945572 appears in two classes as a data-driven split only; it is excluded from the primary logistic model (see main text, Section 3.6).
